# Cognitive Resilience and Vulnerability to Socioeconomic Disadvantage: Predictors Across Individual, Family, School, and Neighborhood Contexts

**DOI:** 10.1111/desc.70105

**Published:** 2025-12-11

**Authors:** Deena Shariq, Rachel R. Romeo, Arianna M. Gard

**Affiliations:** ^1^ Neuroscience and Cognitive Science Program University of Maryland College Park Maryland USA; ^2^ Department of Human Development and Quantitative Methodology University of Maryland College Park Maryland USA; ^3^ Department of Psychology University of Maryland College Park Maryland USA

**Keywords:** cognitive development, early adolescence, ecological systems, person‐centered modeling, resilience, socioeconomic resources

## Abstract

Though much research links socioeconomic disadvantage to cognitive difficulties during adolescence, many youth demonstrate resilience. Person‐centered approaches can be used to quantify this developmental heterogeneity and challenge deficit‐centered frameworks. This study leverages person‐centered and data‐driven methods to quantify and characterize cognitive heterogeneity in a socioeconomically diverse sample of early adolescents from the Adolescent Brain Cognitive Development Study (*N* = 9839; 47.7% female sex; *M*_age_ = 9.90 years; 46.7% White). Four profiles were identified based on their access to socioeconomic resources (SER) and multi‐domain cognitive functioning, including two profiles characterized by moderate‐to‐high SER (74.5%) and two profiles characterized by low SER (25.5%). Among youth in low‐SER environments, 88.6% demonstrated cognitive performance scores similar to youth with moderate‐to‐high access to SER (“cognitive resilience”), whereas 11.4% demonstrated markedly lower performance relative to the other profiles (i.e., 1.3–2.3 SD below the sample mean; “cognitive vulnerability”). Ridge regression identified ecological factors associated with profile membership at the individual level and within family, neighborhood, and school contexts. Suburban residence (odds ratio [OR] = 1.30), advanced pubertal maturity (OR = 1.20), bilingualism (OR = 1.14), and greater caregiver monitoring (OR = 1.10) were most strongly associated with lower‐SER youths’ membership in the resilient versus the vulnerable profile. Results emphasize the need to challenge deficit‐centered frameworks by investigating heterogeneity within profiles of adversity‐exposed youth and identifying context‐specific risk and protective factors.

## Summary

- Over 80% of socioeconomically disadvantaged early adolescents demonstrated cognitive skills on par with their more advantaged peers.
- Among disadvantaged youth, predictors of cognitive resilience spanned individual, family, neighborhood, and school/peer contexts.
- Living in a suburban area, being bilingual, advanced puberty, and greater parental monitoring were associated with a higher likelihood of demonstrating resilience over vulnerability.
- Data‐driven analyses reproduce and extend Masten et al. (2021)’s “short list” of resilience factors for cognitive development in adverse contexts.

## 1 Introduction

Millions of children in the United States experience poverty, live in under‐resourced neighborhoods, and encounter material hardships such as food and healthcare insecurity (Evans 2021). Although much research links childhood socioeconomic disadvantage to cognitive difficulties (Lawson et al. 2018), deficit‐based approaches may overlook variability in children's outcomes (Ellis et al. 2017; Masten 2001) and risk perpetuating stereotypes about historically marginalized communities (Barbarin et al. 2020). The present study uses a population‐based nationwide sample to document this variability and identify ecological factors that predict resilience to socioeconomic adversity, particularly during early adolescence when social‐contextual and biological plasticity is high (Blakemore and Mills 2014).

### 1.1 Cognitive Development in Socioeconomically Disadvantaged Contexts

Socioeconomic disadvantage reflects limited access to socioeconomic resources^1^ (SER) and can be measured at multiple levels of a child's ecosystem (Hyde et al. 2020). Family‐level SER, including household income or caregiver education, reflects access to material resources that meet youths’ basic needs and home‐based cognitive stimulation (Rosen et al. 2018; Hyde et al. 2020), whereas indicators of neighborhood‐level SER indicate exposure to developmental risk and protective factors like violent crime, access to public services, and air pollution (Evans 2021). Neighborhood resources may be particularly salient during early adolescence as youth begin to spend additional time outside of the home with peers and away from caregivers (Blakemore and Mills 2014). Despite the nested and interactive nature of youths’ family and neighborhood environments, each system may have distinct effects on neurocognitive and socioemotional development (Hyde, Gard et al. 2022).

Socioeconomic disadvantage is consistently identified as a risk factor associated with reduced cognitive functioning across domains, including executive function (EF), language, memory, and visuospatial skills (e.g., Hackman et al. 2015; Rosen et al. 2018). For example, low‐SER youth score lower, on average, than their peers on tasks that assess vocabulary, verbal comprehension, and phonetic fluency (Lawson et al. 2018). Several studies also report disparities in EF, a set of distinct but interrelated cognitive processes involved in goal‐directed behavior, including working memory, inhibitory control, and cognitive flexibility (Miyake et al. 2000). In one such study, Last et al. (2018) found that greater access to family SER in childhood was associated with higher performance on six different EF assessments and that this advantage persisted from age 9–25 years. Importantly, childhood cognitive function is a reliable predictor of adult cognitive function (Luna et al. 2004) as well as socioeconomic (e.g., earnings; Furnham and Cheng 2016) and health (e.g., accelerated aging; Schaefer et al. 2016) outcomes in adulthood.

Another set of studies suggests that there may be specificity in which domains of cognitive function are most affected by low SER in early adolescence. In one study, although lower family SER was associated with reduced performance on language, memory, inhibitory control, and working memory tasks, reductions to visuospatial performance were undetected (Farah et al. 2006). Moreover, although socioeconomic disadvantage and other adverse childhood experiences may reduce inhibitory control (Tomlinson et al. 2020), working memory capacity (Nweze et al. 2021), and sustained attention (Razza et al. 2010), several other studies show that procedural memory (Leonard et al. 2015), attention‐shifting, and working memory updating (Young et al. 2022) may be unaffected or even enhanced in low‐SER contexts. These findings align with adaptation‐based theories, which emphasize that specific cognitive abilities may be differentially shaped by exposure to adversity, resulting in the co‐existence of cognitive strengths and weaknesses within individuals (Ellis et al. 2017; Frankenhuis and de Weerth 2013). Proponents of the adaptation‐based framework argue that the value of traits and behaviors is context‐dependent, and cognitive skills that confer advantages in one environment may engender detrimental outcomes in an environment with different demands. For example, in a sample of 681 sociodemographically diverse adolescents (*M*_age_ = 14 years) living in Utah, the disparity in working memory performance between youth with high and low exposure to poverty diminished when task stimuli were ecologically relevant to the participants’ lives (i.e., socially relevant images as opposed to abstract shapes; Young et al. 2022). Such context‐specific enhancements in cognitive functioning might reveal a set of environmental adaptations that could be leveraged to bolster task performance and enhance learning in academic settings (Ellis et al. 2022).

### 1.2 Methodological Challenges, Gaps, and Solutions to Identify Cognitive Resilience to Low SER

Methodological decisions, including the predominant use of variable‐centered frameworks, may inadvertently lead to deficit‐focused interpretations about cognitive development in low SER contexts. Much of the existing literature examines mean‐level associations between SER and cognitive performance. Though such variable‐centered approaches are useful for maximizing statistical power and understanding the relations between two or more specific variables, these frameworks may also overlook within‐group variability. For example, despite replicating an overall negative association between family income and cognitive function in 9–10‐year‐old youth, Ellwood‐Lowe's et al. (2021) graphical depiction of raw data revealed enormous variation in cognitive function at every income level (e.g., many low‐income youth demonstrated above‐average cognitive function). Such diversity suggests there may be subgroups of youth who defy mean‐level trends identified in variable‐centered research.

By contrast, person‐centered approaches, like latent profile analysis (LPA), are designed to capture heterogeneous patterns of predictor‐outcome relations within observed data (McCutcheon 1987). LPA is well‐suited for data‐driven classification of participants who may differ from mean‐level trends, with the added advantage of not relying on arbitrary cutoff points to divide groups. For example, Tyrell et al. (2023) used LPA to identify subgroups of 8–12‐year‐old high‐risk youth using measures of psychosocial functioning and allostatic load. Results indicated a large subgroup of youth who demonstrated resilience across all domains, and smaller subgroups of youth who demonstrated risk in mental and/or physical domains. The person‐centered approach has also been used to identify subgroups of low‐income preschool‐aged youth based on cognitive task performance scores and teacher‐reported behavioral measures (e.g., Bayly and Bierman 2022; Williams and Bentley 2021). However, this line of inquiry has not yet been extended to examine cognitive heterogeneity among youth in later developmental stages.

### 1.3 Risk and Protective Factors for Cognitive Resilience and Vulnerability

In addition to identifying subgroups of youth with distinct SER‐cognition patterns, it is important to pinpoint the environmental factors that covary with resilience and vulnerability in low‐SER contexts. A central goal of resilience research is to identify strengths‐based resources that support children and families in adverse circumstances (Luthar et al. 2000; Masten et al. 2021). Indeed, resilience research originated from studies demonstrating heterogeneity in the childhood adversity effects on psychopathology, with researchers finding that youth who fared better often had enhanced access to supports and resources (Masten 2001). In recent decades, the study of resilience has expanded to examine risk and protective factors for nonclinical outcomes, including EF (e.g., Fields et al. 2021), and academic success (e.g., Jagannathan et al. 2023).

For youth living in socioeconomically disadvantaged environments, confronting additional risk factors may directly or indirectly magnify cognitive difficulties (e.g., Walker et al. 2011), whereas access to protective factors may reduce stress burden (e.g., Tyrell et al. 2023) and provide additional sources of cognitive stimulation (e.g., Romeo et al. 2018) or social capital (e.g., Ashtiani and Feliciano 2018). Risk and protective factors exist across ecological systems (Bronfenbrenner 1977; Masten et al. 2021; Hyde et al. 2020), from proximal (e.g., within individuals, families) to more distal (e.g., schools, neighborhoods). Research examining resilience factors in a wide range of adverse contexts consistently identifies a set of influential ecological factors, compiled in Masten's “short list” (Masten 2021). Here, we focus on resilience‐promoting factors across four ecological contexts—individual, family, school, and neighborhood—and augment the “short list” with factors specifically tied to performance on cognitive measures (Rakesh, Lee et al. 2025, Rakesh, McLaughlin et al. 2024, Rakesh, Sadikova et al. 2024).

#### 1.3.1 Individual

At the individual level, mental health, physical health, and linguistic experiences are associated with cognitive development. For example, youth who report greater anxiety are more likely to exhibit impairments in working memory capacity (Moran 2016), whereas externalizing behaviors, such as impulsivity and rule‐breaking, are more closely tied to individual differences in inhibitory control (Schoemaker et al. 2013). Reward sensitivity, also linked to psychopathology through motivational processes, is tied to individual differences in inhibitory control (Prabhakaran et al. 2011) and working memory (Gray and Braver 2002; Unsworth et al. 2009). In addition, pubertal timing has been linked to changes in brain networks facilitating EF abilities (Hackman et al. 2015), and accelerated pubertal maturation was associated with lower performance on EF tasks (Stumper et al. 2020). Finally, a cadre of studies has explored the associations between bilingualism and EF, although findings are mixed (for a meta‐analysis, see Gunnerud et al. 2020). Bilingualism may benefit socioeconomically under‐resourced youth by enhancing EF broadly (Grote et al. 2021) or in selective domains (e.g., inhibitory control, verbal working memory, and visual working memory; Engel de Abreu et al. 2012; Ware et al. 2020).

#### 1.3.2 Family Context

The Family Stress (FSM) model (Conger et al. 2010) argues that socioeconomic disadvantage impacts children via its effects on caregivers, with mediators including caregiver psychopathology and family conflict. Indeed, compared to socioeconomically advantaged caregivers, caregivers with low SER report greater internalizing symptoms and chronic stress (Ursache et al. 2017), which, in turn, is associated with reductions in children's EF (e.g., Jensen et al. 2014). Additionally, meta‐analyses (e.g., Rodrigues et al. 2021) indicate that home environments characterized by high positive (e.g., responsiveness, warmth) and low negative (e.g., detachment, conflict) caregiving behaviors are linked to children's improved performance on EF tasks. Caregiver distress can also confer risk for problematic alcohol/substance use, a known adverse childhood experience associated with youth attention problems (Torvik et al. 2011) and lower overall IQ scores (Poon et al. 2000). Finally, caregivers' cognitive skills are associated with their children's cognitive skills through shared genetics and impacts on parenting (Byford et al. 2012).

#### 1.3.3 School and Peer Context

Social cues from peers and classmates become more salient during early adolescence (Blakemore and Mills 2014). Schools provide spaces for youth to socialize with peers and form friendships, which can function as sources of cognitive learning and emotional support (Hartup 1998). At the same time, peer victimization and rejection are associated with reduced EF (Holmes et al. 2016). Schools also facilitate relationships between youth and unrelated adults, and the quality of interactions between students and teachers is linked to children's working memory and inhibitory control capacity (Vandenbroucke et al. 2018). Additional school‐level risk and protective factors for cognitive development include school climate, a construct encompassing youths’ perceptions of academic support, safety, social connectedness, and organization (Thapa et al. 2013). The advantage of having a positive school climate for EF is enhanced among socioeconomically disadvantaged youth, pointing to its role as a resilience‐promoting factor (Piccolo et al. 2019). Finally, student engagement in school activities predicts adolescents’ performance on sustained attention assessments (Hobbiss and Lavie 2024), and rates of disengagement are higher among low‐SER youth (Tomaszewski et al. 2020).

#### 1.3.4 Neighborhood Context

Lastly, living in a neighborhood characterized by high crime and/or perceived danger has been linked to lower EF performance (e.g., Meredith et al. 2022), partly due to increases in physiological and psychological distress (Hyde et al. 2020). However, neighborhoods characterized by cohesion and trust are shown to buffer the negative effects of exposure to violent crime on youth mental health (Ozer et al. 2017), though few scholars have examined the plausible promotive effect of cohesive neighborhoods for children's cognitive function. In parallel to the social dimensions (i.e., danger, cohesion) of the neighborhood environment, physical attributes of a community, including greenspace, crowding, and pollution, also serve as risk and promotive factors for cognitive development by shaping the ways that youth interact with their surroundings (Nordbø et al. 2020). For example, youth who live in neighborhoods with greater amounts of greenspace demonstrate advantages in attentional capacity, working memory, and inhibitory control (see Vella‐Brodrick and Gilowska 2022 for a review). On the contrary, high rates of residential crowding and noise (e.g., from aircrafts or automobile traffic) are associated with impairments in reading and numeracy skills (Solari and Mare 2012). Cognitive risks can also be introduced through environmental pollutants (e.g., lead, particulate matter), which disproportionately impact neighborhoods with higher proportions of Black families and families with less access to SER (Jbaily et al. 2022). For example, lead exposure is associated with reduced performance on EF, vocabulary, and processing speed (Marshall et al. 2021), and air pollution has been linked to impairments in working memory, short‐term memory, and attentional processes (Lopuszanska and Samardakiewicz 2020). Finally, neighborhood‐level risk and promotive factors for cognitive functioning could also be explained by the urbanicity of one's neighborhood (Tine 2019), though research linking neighborhood factors to cognitive development has largely focused on impoverished urban contexts.

### 1.4 The Present Study

This study pairs an adaptation‐based framework with person‐centered and data‐driven analyses to identify patterns and predictors of SER‐dependent cognitive performance in a large population‐based study of early adolescents. First, estimates of youths’ socioeconomic conditions (i.e., family and neighborhood SER) and domain‐specific cognitive performance (i.e., verbal and reasoning [VR], speed and flexibility [SF], and memory scores) were used to identify subgroups of early adolescents reflecting joint patterns of cognitive functioning and socioeconomic risk (**Aim 1**). We hypothesized the emergence of several “typical” profiles wherein relative SER and cognitive function corresponded (e.g., high SER and high cognitive performance, low SER and low cognitive performance) in addition to one or more “atypical” profiles wherein relative SER and cognitive abilities diverged (e.g., high SER and low cognitive performance). Specifically, we predicted the emergence of a “resilient” profile characterized by youth with low SER and average‐to‐high cognitive functioning. We also allowed for the emergence of additional profiles differentiated by domain‐specific cognitive performance, such as a profile with low SER and a mix of high and low cognitive domain scores.

Next, we examined whether latent profiles differed in key sociodemographic constructs and school‐based indicators of functioning (e.g., grades, grade retention; **Aim 2**). Given well‐documented racial disparities in access to family and neighborhood SER (Reeves et al. 2016), we anticipated disproportionately higher proportions of Black and Hispanic youth in low‐SER profiles compared to high‐SER profiles. We also predicted higher rates of grade retention and lower grade point averages (GPA) in profiles characterized by clear cognitive vulnerabilities compared to profiles with higher cognitive performance.

Lastly, we sought to distinguish latent profiles of socioeconomically disadvantaged youth who varied in cognitive performance by examining risk and protective factors across multiple ecological contexts (i.e., individual, family, neighborhood, and school/peer; **Aim 3**). Multinomial logistic ridge regression (Simon et al. 2011) was employed to identify the relative influence of 55 ecological variables on youths’ odds of profile classification. Relying on data‐driven variable selection, ridge regression allowed us to include more predictors without sacrificing degrees of freedom, mitigating concerns about bias in parameter selection and multicollinearity (Van Lissa 2023). We hypothesized that factors shown to be associated with positive cognitive developmental outcomes (e.g., caregiver warmth) would increase the odds of being classified into a cognitive resilience profile, whereas previously identified risk factors (e.g., neighborhood violence) would decrease the odds of being classified into a cognitive resilience profile. We further expected risk and protective factors to be distributed across all levels of ecological influence (Masten et al. 2021; Hyde et al. 2020).

## 2 Methods

### 2.1 Participants

Given the magnitude of predictors and likely small (but cumulative) effect sizes, a large dataset was needed to evaluate the proposed aims. The present study leveraged baseline data from the Adolescent Brain Cognitive Development Study (ABCD Study), a longitudinal population‐based study of 11,875 eligible 9–10‐year‐olds in the United States (Garavan et al. 2018). Participants were recruited using clustered probability sampling from public and private schools within 21 catchment areas near study sites. Multiple children from each household were eligible to participate (see Supporting Methods for exclusion criteria). All aspects of the ABCD Study protocol were approved by each study site's Institutional Review Board. Participating caregivers were administered informed consent and provided caregiver consent for participating minors. Data were downloaded from the fourth curated public release (https://doi.org/10.15154/1523041) from the NIMH Data Archive collection #2573 (DUA #3067).

Participants missing baseline data for all cognitive assessments (*n* = 13) or all SER indicators (*n* = 4) were excluded. Due to the exact overlap of SER within families, analyses excluded one random child per family in which multiple children participated (*n* = 2019). The analytic sample included 9839 youth (*n* = 4693; 47.7% female sex; *M*_age_ = 9.90 years, SD = 0.62, Range = 8.92–11.08 years). Although the analytic sample significantly differed from the full sample along key demographic characteristics, effect sizes were small (i.e., maximum Cramer's *V* = 0.02; Table S1). The racial‐ethnic makeup of the analytic sample included 200 (2.0%) Asian youth, 908 (9.2%) Biracial or Multiracial youth, 1261 (12.8%) Black youth, 1908 (19.4%) Hispanic/Latinx youth, 4592 (46.7%) White youth, 34 (0.4%) Native American/ Alaskan/Hawaiian youth, 7 (0.1%) Pacific Islander youth, and 929 (9.4%) youth of an unspecified or “other race” (see Table S2 for a description of how sociodemographic variables were coded). Within the analytic sample, 59.6% of youth had a caregiver with a Bachelor's degree or higher (*M*_years of education_ = 16.79, SD = 3.16), and 15.1% lived in households with a combined income equal to or below the federal poverty line (*M*_income‐to‐needs ratio_ = 3.74, SD = 2.50; see Supporting Methods for all descriptive statistics).

### 2.2 Measures

#### 2.2.1 Cognitive Performance

Youth cognitive performance across three broad domains was assessed with latent factor scores obtained from a confirmatory factor analysis (CFA). The CFA model included a total of 10 task performance indicators from a battery of neurocognitive assessments administered in the ABCD Study, which took ∼70 min and was delivered in English (Luciana et al. 2018). Seven neurocognitive assessments were collected using the NIH Toolbox (NIHTB; Weintraub et al. 2013): (1) the NIHTB Picture Vocabulary Task to capture language and verbal performance; (2) the NIHTB Oral Reading Recognition Task, which measures reading skills; (3) the NIHTB Pattern Comparison Processing Speed Test, to capture rapid visual information processing; (4) the NIHTB List Sorting Working Memory Test, indexing working memory ability; (5) the NIHTB Picture Sequence Memory Test measuring episodic memory; (6) the NIHTB Flanker Task to capture inhibitory control; and (7) the NIHTB Dimensional Change Card Sort Task which measures cognitive flexibility. Lastly, (8) short‐term and (9) long‐term memory retention were captured by the Rey Auditory Verbal Learning Test (RAVLT; Schmidt 1996), and (10) nonverbal abstract reasoning was assessed through the Matrix Reasoning subtest from the Wechsler Intelligence Test for Children‐V (WISC‐V; Wechsler 2014). Although EF was not modeled as a separate latent construct, core components of EF, including working memory, inhibitory control, and cognitive flexibility, were represented across the tasks included in the CFA (Best and Miller 2010; Miyake et al. 2000). See Supporting Methods for full task descriptions and sample descriptive statistics.

**FIGURE 1:**
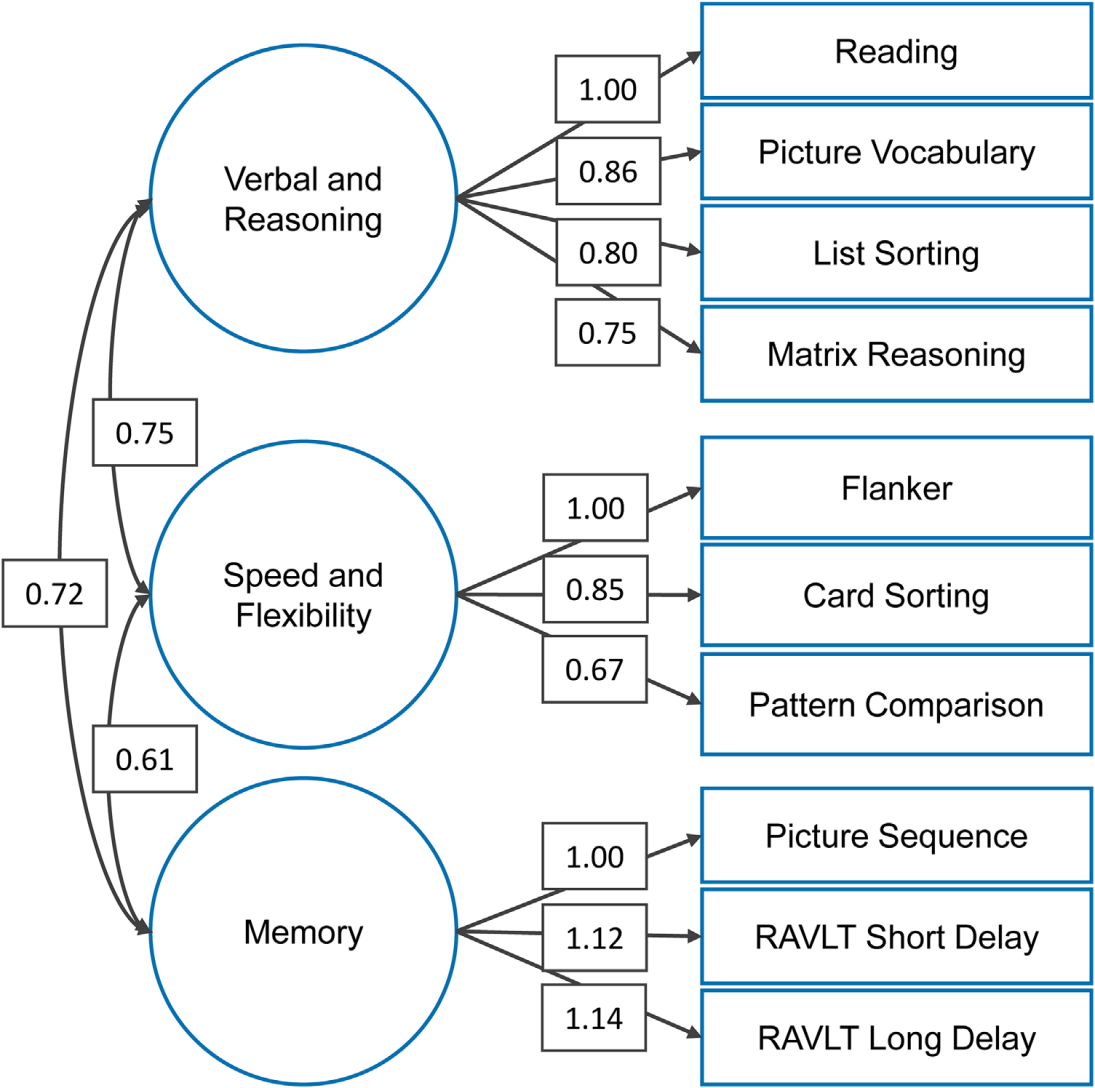
Factor structure of cognitive performance. *Note*: Total *N* = 9839, comparative fit index (CFI) = 0.925, Tucker‐Lewis index (TLI) = 0.904, root mean square error of approximation (RMSEA) = 0.082. RAVLT = Rey auditory verbal learning task. Standardized estimates are displayed.

The latent factor structure of cognition was estimated using CFA. For NIHTB tasks, uncorrected, raw scores were used. Each measure was standardized via z‐score transformation (i.e., *M* = 0, SD = 1), data were clustered by study site, and latent factors were allowed to covary. CFA was conducted in Mplus version 8.0 (Muthén and Muthén 1998–2017) using the Maximum Likelihood Estimator with Robust Standard Errors (MLR). Model fit was considered acceptable if indicator loadings were >0.3, the Root Mean Square Error of Approximation (RMSEA) was <0.08, and the Comparative Fit Index (CFI) was >0.90. Although this approach was based on Sripada et al. (2021), who identified a three‐factor bifactor model with domain‐specific latent factors of verbal/spatial, speed/flexibility, and memory, and an overarching general cognitive ability factor, our re‐analysis of the complete baseline data with appropriate nesting by study site resulted in a three‐factor model of youth cognitive performance with acceptable model fit (CFI = 0.925; TLI = 0.904; RMSEA = 0.082; Figure 1). The VR latent factor explained significant variation in the WISC‐V Matrix Reasoning Test and the NIHTB Oral Reading Recognition, Picture Vocabulary, and List Sorting Tasks; the SF latent factor explained significant variation in the NIHTB Flanker, Card Sort, Pattern Comparison Processing Speed Tasks; and the memory (MEM) latent factor explained significant variation in the NIHTB Picture Sequence Test and long‐ and short‐term memory scores from the RAVLT. See Supporting Methods and Figure S1 for factor score validation. Participant‐level factor scores for VR, SF, and MEM were extracted for further analysis, with higher values reflecting greater cognitive performance in each domain.

#### 2.2.2 SER

**FIGURE 2:**
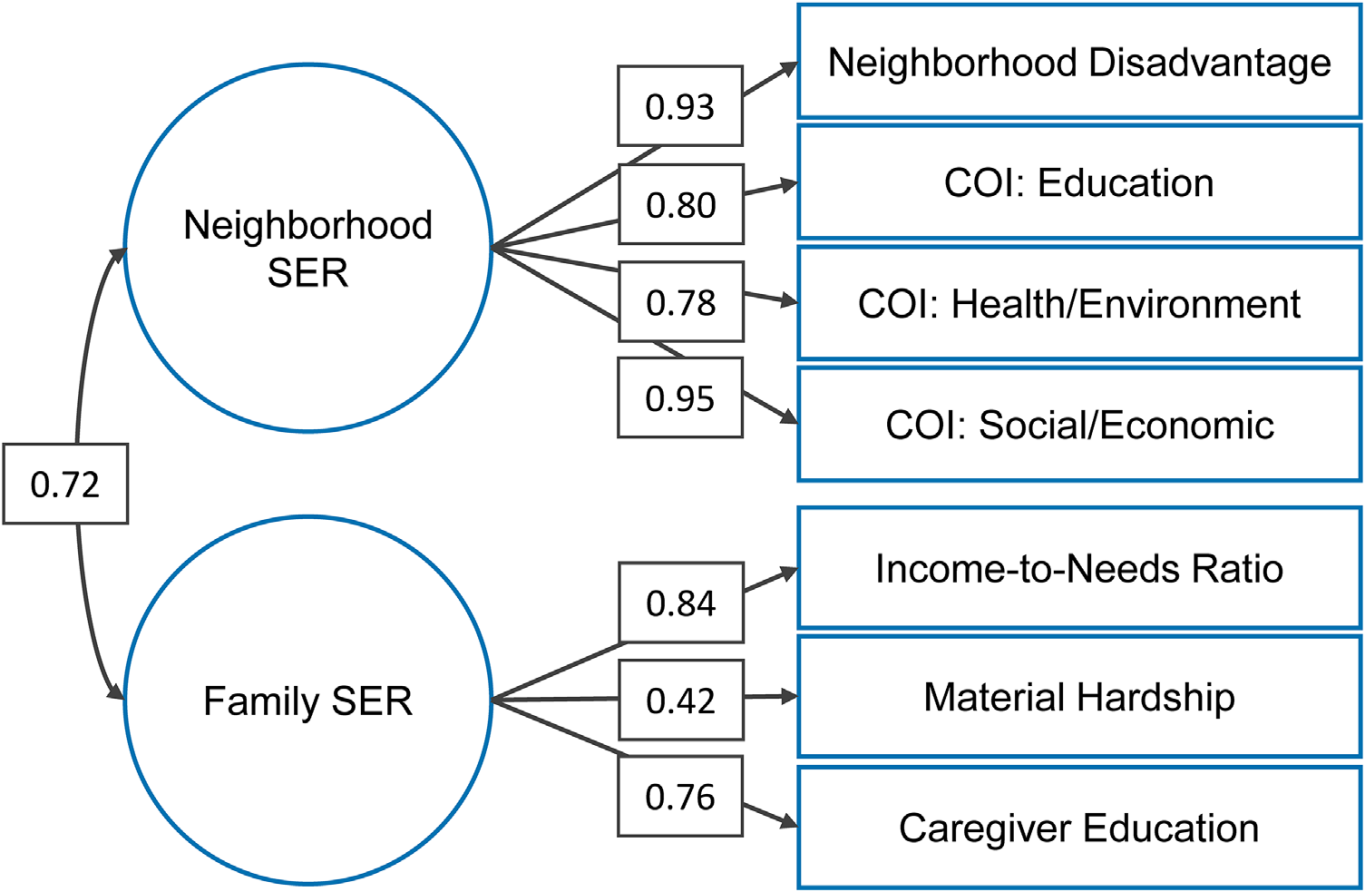
Factor structure of socioeconomic resources. *Note*: Total *N* = 9839, COI = child opportunity index, comparative fit index (CFI) = 0.976, Tucker‐Lewis index (TLI) = 0.961, root mean square error of approximation (RMSEA) = 0.025. SER = socioeconomic resources. Standardized estimates are displayed.

Youths’ access to SER in the family and neighborhood contexts was assessed with latent factor scores derived using CFA. The CFA model included four indicators fit onto the latent construct of neighborhood SER, including (1–3) the social/economic, health/environment, and education subscales of the Child Opportunity Index, a zip‐code level measure of neighborhood resources and conditions associated with children's healthy development (Acevedo‐Garcia et al. 2020); and (4) a neighborhood socioeconomic disadvantage score measured using nine items (e.g., percentage of residents with a Bachelor's degree or higher) at the Census‐tract‐level (Taylor et al. 2020). Three indicators were fit onto the latent construct of family SER, including parent‐reported (1) caregiver education, quantified as the highest level of education attained by the youth's primary caregiver(s); (2) household income‐to‐needs ratio, calculated by dividing the median of the caregiver‐reported household income range by the 2017 federal poverty line for their household size; and (3) material hardship, measured as a sum score of 7 dichotomous (Yes = 1) items from the Material Hardship questionnaire. All measures were standardized via z‐score transformation, data were clustered by study site, latent factors were allowed to covary, and the MLR estimator was employed. The material hardship and neighborhood disadvantage variables were multiplied by −1 so that more positive values corresponded with less hardship or disadvantage. The two‐factor model of SER fit the data well (CFI = 0.976; TLI = 0.961; RMSEA = 0.025; Figure 2), with latent factors explaining significant variation in corresponding indicator variables. Participant‐level latent factor scores for family SER and neighborhood SER were extracted for further analyses, with higher values reflecting greater SER. In contrast to principal component analysis or index‐based methods, CFA allowed us to test a theory‐driven, two‐factor structure in which family and neighborhood SER were modeled as distinct but correlated constructs, reflecting our conceptualization of SER as a multidimensional contextual influence rather than a single formative index. See Supporting Methods for sample descriptive statistics.

#### 2.2.3 Predictors of Profile Membership

The extensive collection of youth‐ and caregiver‐reported measures, and administrative data in the ABCD Study were used to predict cognitive risk and resilience to low SER based on our expanded “short list” of resilience factors (Masten et al. 2021). Fifty‐five predictors were queried, spanning individual (*n* = 22), family (*n* = 18), school (*n* = 4), and neighborhood (*n* = 11) ecological systems. Constructs were chosen based on previous associations with cognitive development in meta‐analyses and systematic reviews (e.g., Lopuszanska and Samardakiewicz 2020; Rakesh et al. 2025; Rodrigues et al. 2021) and/or conceptualizations as broad risk or protective factors (e.g., Gartland et al. 2019; Masten 2001; Walker et al. 2011). Variables are described briefly here, with extensive measurement details and descriptive statistics in Table S3. Individual‐level predictors included eight population‐normed subscales of caregiver‐reported youth mental health, youth‐reported psychosis symptoms, five measures of youth impulsivity (e.g., sensation seeking, negative urgency), youths’ behavioral avoidance and behavioral reward sensitivity, caregiver‐reported youth prosocial behavior, youth pubertal maturation (measured by averaging child‐ and caregiver‐reports), youth body mass index (BMI), age in months, exposure to mature content in media (e.g., R‐rated video games), and bilingualism. Family‐level predictors included eleven indicators of primary caregiver self‐reported mental health (e.g., anxiety/depression symptoms), caregiver history of alcohol or drug abuse, family history of completed suicide, and youth‐reported family conflict, caregiver warmth, and parental monitoring. School/peer‐level predictors included youth self‐reported disengagement from school, positive attitudes about school involvement, positive perceptions of the school environment (i.e., safety, availability of resources, and supportiveness), and the number of “close friends”. Neighborhood‐level predictors included youth perception of neighborhood safety, county‐level crime rates, estimated exposure to nitrous dioxide (i.e., 1 km^2^ around household address), and household distance (meters) to major roads or highways. Administrative data harmonized to geocoded participant addresses were used to measure residential crowding, median home value, median rent or mortgage, estimated risk of lead exposure, and Census tract urbanicity classification (i.e., urban, suburban, and rural).

### 2.3 Statistical Analysis

LPA was used to identify homogenous subgroups of youth based on estimated SER (i.e., family and neighborhood SER latent factor scores) and youth cognitive performance (i.e., VR, SF, and memory latent factor scores). We chose to model SER and cognitive function jointly within the LPA to capture naturally occurring patterns of contextual adversity and cognitive competence. LPA was performed in Mplus v.8 (Muthén and Muthén 1998–2017) with the MLR estimator and TYPE = COMPLEX to allow for clustering by study site. As profile membership is an unknown latent variable, the number of profiles was determined by evaluating the model fit of a one‐profile solution and iteratively increasing the number of profiles until model fit depreciated (Collins and Lanza 2009). Models were estimated with multiple random start values to minimize the risk of local maxima and ensure the best log‐likelihood value was replicated. Model fit statistics included the Bayesian Information Criterion (BIC) and the Akaike Information Criterion (AIC), for which lower scores represent better model fit; and the entropy value, for which higher scores (i.e., closer to one) indicate greater classification accuracy while values under 0.7 are indicative of uncertain cluster assignment (Collins and Lanza 2009). Starting values were varied, and the highest log‐likelihood was replicated at least twice, indicating a global maximum rather than a local solution. Models were re‐run without clustering by study site to assess model fit via the Lo‐Mendell Rubin test, which compares the log‐likelihood value between neighboring k‐profile models (e.g., two‐profile versus three‐profile); *p* values of <0.05 indicate a statistically significant improvement in model fit for the inclusion of one more profile. In addition to statistical indices, model parsimony (i.e., profile size; meaningful difference in mean SER and cognitive performance values) was used to select the best‐fitting model in terms of the number of profiles.

Following LPA, we conducted post‐hoc tests of measurement invariance to evaluate whether the identified profile structure generalized across four demographic variables, including race‐ethnicity, gender, family structure, and country of origin (Lanza and Bray 2010). Unconstrained (configural) and constrained (scalar) models were estimated for each grouping variable, and model fit was assessed based on changes in AIC, BIC, and log‐likelihood. Given that measurement invariance was evaluated after profile solutions and group comparisons were finalized, observed differences in profile composition across demographic groups should be interpreted cautiously.

Multinomial logistic ridge regression (Simon et al. 2011) was used to characterize which of the 55 plausible risk and promotive factors predicted profile membership. Ridge regression, also called L2 regularization, applies a penalty term to shrink less impactful coefficients closer to zero without fully removing them from the model. This technique is particularly appropriate for high‐dimensional data structures with substantial multicollinearity between predictors, as it reduces bias in parameters while retaining all predictors (Simon et al. 2011). Ridge regression was selected over alternative regularization methods, such as LASSO or elastic net, given our goal of stabilizing coefficient estimates without excluding predictors. In contrast to LASSO (L1) regularization, in which coefficients can be shrunk to zero, and elastic net regularization, which incorporates both L1 and L2 penalties and can also remove predictors, ridge regression preserves all variables, including those with small effect sizes. Thus, ridge regression enables a data‐driven, machine learning approach to systematically evaluate predictor contributions and refine theoretical models in developmental psychology (Van Lissa 2023).

Analyses were conducted in R Statistical Software version 4.2.2 (R Core Team 2023) using the *glmnet* package for logistic ridge regression analysis (Friedman et al. 2010). All continuous predictors were winsorized to ± 3 SD from the mean, and missing values were imputed. To estimate stable model parameters, we implemented a bootstrapping procedure combined with cross‐validation. Specifically, 1000 bootstrapped samples were drawn from our data (with replacement), and within each bootstrap sample, 10‐fold cross‐validation was used to determine the optimal penalty parameter (λ) that minimized the misclassification rate across validation subsets (Chatterjee and Lahiri 2011). Next, the multinomial ridge regression model was fit to the bootstrap sample using this optimized λ value. Each profile had a unique regression equation with 62 beta coefficients (i.e., one per continuous predictor, one per level of a categorical predictor, and one for the intercept). This procedure generated a distribution of coefficient estimates across bootstrap samples for each profile‐predictor pairing. From these distributions, we estimated the log odds estimate (i.e., the 50^th^ percentile) and 95% confidence interval (CI; i.e., 2.5^th^ and 97.5^th^ percentiles) for each predictor and latent profile (Chatterjee and Lahiri 2011).

To examine how ecological predictors contributed to youths’ odds of being a member of one profile over another, we derived odds ratios (OR) by calculating the exponentiated difference in log odds estimates for each of the 62 coefficients, across all possible profile pairs. Here, ORs represent the degree to which a 1‐unit increase in the predictor variable (or the endorsement of a binary predictor variable) increases or decreases the odds of being in one profile versus another. An OR was determined to be significant if its 95% CI did not include 1, which would indicate no change in odds with changes made to the predictor. Given our focus on understanding risk and protective factors for youth with low SER, comparisons described in the main text are limited to profiles characterized by socioeconomic disadvantage.

### 2.4 Transparency and Openness

We describe all inclusion and exclusion criteria for the analytic sample, all measures, and provide descriptive statistics for all measures here and in the Supporting Materials. The data necessary to reproduce the analyses presented here are publicly accessible through the ABCD Study and can be accessed at the following URL: https://doi.org/10.15154/1523041. Analyses were not preregistered. The analytic code necessary to reproduce the analyses from raw data is publicly accessible (https://osf.io/j3rvs/).

## 3 Results

### 3.1 Aim 1: Profiles of SER‐Dependent Cognitive Functioning

First, LPA was used to identify profiles of youth reflecting joint cognitive‐SER patterns. Models of one to five profiles each were tested, with the four‐profile solution demonstrating the best balance of fit and interpretability (AIC = 86,377.49; BIC = 86,758.78; Entropy = 0.703; see Tables S4 and S5 for full model fit statistics). Although the addition of a fifth profile slightly improved the log likelihood, BIC, and AIC, these differences were minimal, and the fifth profile did not show a substantively distinct pattern of SER or cognitive scores. Moreover, the entropy and average latent class probabilities declined in the five‐profile solution (entropy <0.7, some probabilities <0.7), indicating reduced classification accuracy (Andruff et al. 2009). Thus, the four‐profile solution was selected to maximize statistical fit, parsimony, and conceptual clarity.

**FIGURE 3:**
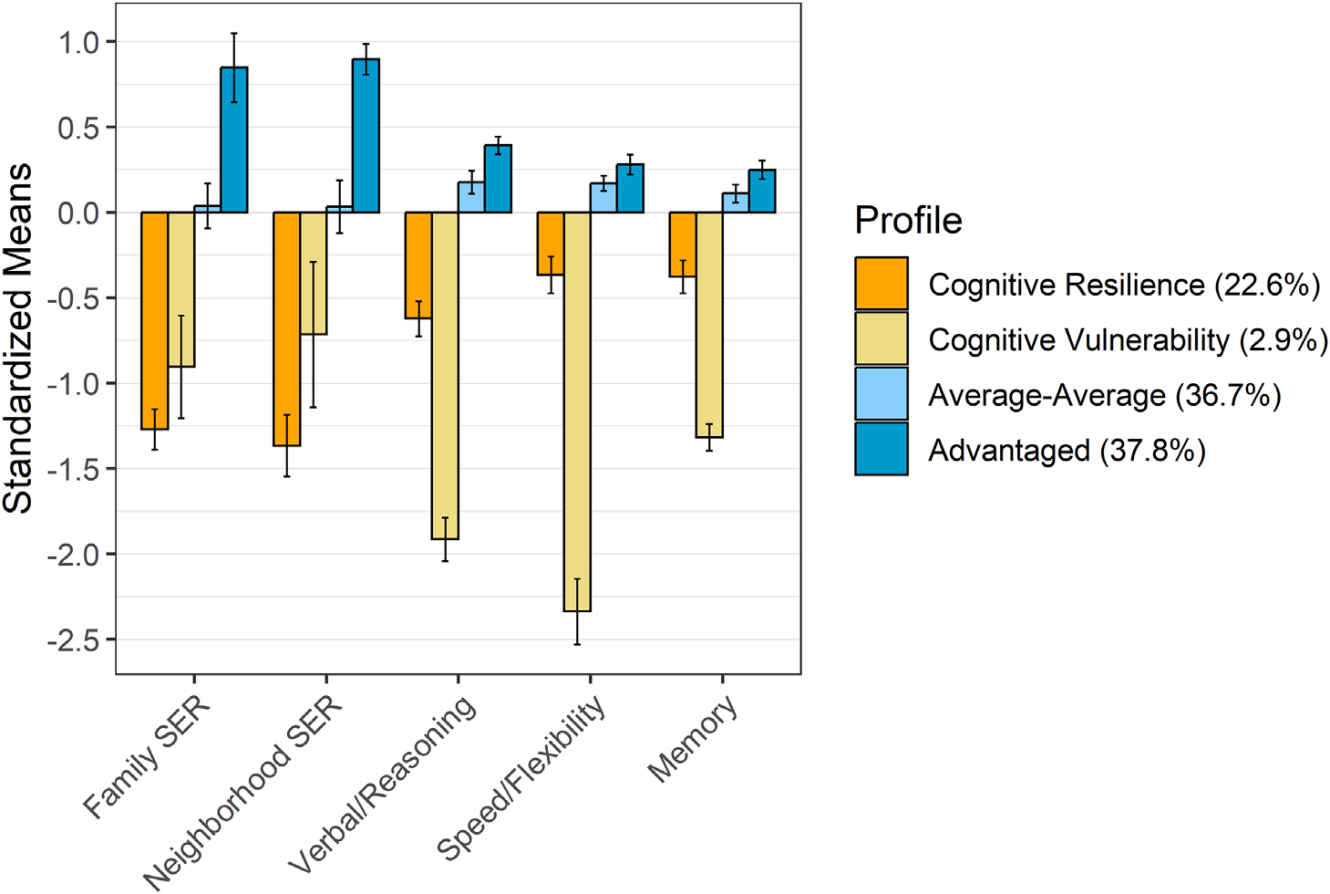
Standardized means for the four‐profile solution to latent profile analysis domain‐specific cognitive performance and socioeconomic resource (SER) scores. *Note*: Total *N* = 9839, cognitive resilience *n* = 2225, cognitive vulnerability *n* = 287, average–average *n* = 3611, advantaged *n* = 3716. Model fit statistics for the four‐profile solution were acceptable (AIC = 86377.49, BIC = 86758.78, Entropy = 0.703). All profiles statistically differed in family SER (*F* (3) = 8133, *p* < 0.001, *η* ^2^ = 0.71), neighborhood SER (*F* (3) = 12780, *p* < 0.001, *η*2 = 0.80), verbal and reasoning (*F* (3) = 1442, *p* < 0.001, *η* ^2^ = 0.31), speed and flexibility (*F* (3) = 1326, *p* < 0.001, *η* ^2^ = 0.29), and memory (*F* (3) = 491.5, *p* < 0.001, *η* ^2^ = 0.13). Mean family SER, neighborhood SER, and domain‐specific cognitive performance scores are plotted for each profile. Bars represent each profile's standardized mean score per variable (i.e., [profile‐average score—full sample average score] ÷ full sample standard error). Error bars represent upper and lower bounds of the 95% confidence interval (i.e., profile‐level mean ± 1.96*profile‐level standard error). SER = Socioeconomic resources.

All profiles statistically differed in family SER (*F*(3) = 8133, *p* < 0.001, *η* ^2^ = 0.71), neighborhood SER (*F*(3) = 12780, *p* < 0.001, *η* ^2^ = 0.80), VR (*F*(3) = 1442, *p* < 0.001, *η* ^2^ = 0.31), SF (*F*(3) = 1326, *p* < 0.001, *η* ^2^ = 0.29), and memory (MEM; *F*(3) = 491.5, *p* < 0.001, *η* ^2^ = 0.13). Figure 3 displays each profile's standardized mean scores for each latent indicator of SER and cognitive performance (see Table 1 and Table S6 for profile‐level averages of unstandardized socioeconomic variables and cognitive test scores, respectively). Resilience was conceptualized as simultaneously having relatively high cognitive functioning in one or more domains despite low access to SER (Masten et al. 2021).

**TABLE 1:** Profile‐level descriptive statistics for indicators of SER.

	Advantaged *M* (SD)	Average–average *M* (SD)	Cognitive resilience *M* (SD)	Cognitive vulnerability *M* (SD)	Test statistics and effect size
Income‐to‐needs ratio	5.56 (1.86)	3.41 (2.18)	1.16 (0.97)	1.50 (1.41)	*F* (37,804) = 2130,
					*p* < 0.001,
					*η* ^2^ = 0.45
Caregiver education	18.78 (1.25)	17.24 (2.08)	14.26 (2.65)	15.04 (2.52)	*F* (38,516) = 2155,
					*p* < 0.001,
					*η* ^2^ = 0.43
Material hardship	0.06 (0.37)	0.47 (1.05)	1.14 (1.56)	0.78 (1.17)	*F* (38,578) = 464.1,
					*p* < 0.001,
					*η* ^2^ = 0.14
COI: social/economic	87.12 (10.19)	55.28 (19.07)	16.15 (12.17)	40.32 (27.32)	*F* (37,959) = 8553,
					*p* < 0.001,
					*η* ^2^ = 0.76
COI: education	83.08 (14.89)	58.65 (24.01)	24.28 (19.98)	43.44 (27.65)	*F* (37,959) = 3247,
					*p* < 0.001,
					η^2^ = 0.55
COI: health/environment	80.14 (17.43)	56.62 (24.21)	22.53 (19.86)	41.36 (30.22)	*F* (37,959) = 2841,
					*p* < 0.001,
					*η* ^2^ = 0.52
Neighborhood disadvantage	−0.65 (0.28)	−0.02 (0.42)	1.18 (0.72)	0.50 (0.97)	*F* (38,266) = 5698,
					*η* ^2^ = *p* < 0.001,
					0.67

*Note*: Total *N* = 9839, cognitive resilience *n* = 2225, cognitive vulnerability *n =* 287, average–average *n =* 3611, advantaged *n* = 3716. Caregiver education was measured as years of education for the caregiver with the highest educational attainment. Profiles differed across all SER indicator variables, except for CV and CR, where there were nonsignificant differences in the income‐to‐needs ratio (*p* = 0.09). Significance was tested through ANOVA tests.

Abbreviations: COI = child opportunity index, *M* = mean, SD = standard deviation.

First, a large profile (*n* = 3716 [37.8% of youth]), characterized by the least socioeconomic disadvantage (i.e., neighborhood SER = 0.90 SD above the sample mean; family SER = 0.85 SD above the sample mean) and the highest cognitive performance (i.e., VR = 0.39 SD above the sample mean; SF = 0.28 SD above the sample mean; MEM = 0.25 SD above the sample mean), was named “Advantaged” (ADV). A similarly large profile (*n* = 3611 [36.7% of youth]), characterized by average SER (i.e., family and neighborhood SER statistically indistinguishable from overall sample means) and nearly average cognitive performance scores (i.e., VR = 0.18 SD above the sample mean; SF = 0.17 SD above the sample mean; MEM = 0.11 SD above the sample mean), was named “Average‐Average” (AVG). A small profile (*n* = 287 [2.9% of youth]), characterized by high socioeconomic disadvantage (i.e., neighborhood SER = 0.71 SD below the sample mean; family SER = 0.90 SD below the sample mean) and the lowest cognitive performance in the sample (i.e., VR = 1.91 SD below the sample mean; SF = 2.34 SD below the sample mean; MEM = 1.32 SD below the sample mean), was named “Cognitive Vulnerability” (CV). The identification of the first three profiles supports our hypothesis that there would be groups of youth whose cognitive abilities and access to SER are aligned. The last identified profile (*n* = 2225 [22.6% of youth]), characterized by the greatest level of socioeconomic disadvantage (i.e., neighborhood SER = 1.37 SD below the sample mean; family SER = 1.27 SD below the sample mean) and cognitive performance within one SD of the sample mean, suggesting adaptive cognitive functioning despite risk (i.e., VR = 0.62 SD below the sample mean; SF = 0.37 SD below the sample mean; MEM = 0.38 SD below the sample mean), was named “Cognitive Resilience” (CR). Examining the indicators that comprised the neighborhood‐ and family‐level SER latent factor (Table 1), CR youth experienced the greatest degree of socioeconomic disadvantage (i.e., least years of caregiver education, greatest material hardship, greatest neighborhood disadvantage, and lowest Child Opportunity Index scores) compared to all other profiles. Together, these results support our first hypothesis; we identified three “typical” profile wherein cognitive function and access to SER was aligned (i.e., low, average, high), as well as a unique profile characterized by low SER and average cognitive function.

To validate our characterization of the latent profiles’ cognitive performance, we examined profile‐level WISC‐V Matrix Reasoning scores, the only available measure normed in nationally representative samples with scoring guidelines linked to IQ (Kaufman et al. 2015). On average, youth characterized as CR, ADV, and AVG had scaled Matrix Reasoning scores within the “Average” score range (i.e., 8–11), demonstrating cognitive functioning within the typical range of development. In contrast, CV youths had an average scaled WISC‐V score within the “Low Average” score range (i.e., 6–7). See the Supporting Methods and Table S7 for a description of additional validation procedures.

### 3.2 Aim 2: Sociodemographic Composition of SER‐Dependent Cognitive Profiles

Next, we assessed differences in sociodemographic composition between profiles using chi‐square tests of independence for categorical variables and one‐way ANOVA for continuous variables (Table 2). We prioritized effect sizes over *p*‐values when interpreting profile differences (e.g., Cramer's *V* for Chi‐square Tests and *η* ^2^ for ANOVA). To highlight the most meaningful patterns, we interpreted associations of at least a small effect size (i.e., *V* > 0.1, *η* ^2^ > 0.01; Cohen 1988), while reporting all effect sizes in Table 2 for transparency. The interpretive threshold for Cramer's *V* was refined post hoc (from *V* ≥ 0.2 to *V* ≥ 0.1) because smaller *V* values can represent meaningful effects in larger contingency tables (Bakker et al. 2019); readers may consider this interpretive change in evaluating profile differences of *V* < 0.20 (Lakens 2024).

**TABLE 2:** Profile‐level differences in sociodemographic constructs and school‐based indicators of functioning.

	Advantaged	Average–average	Cognitive resilience	Cognitive vulnerability	
	% or *M* (SD)	% or *M* (SD)	% or *M* (SD)	% or *M* (SD)	Test statistic and effect size
*Sociodemographic constructs*					
**Age (years)**	9.92 (0.62)	9.92 (0.62)	9.87 (0.61)	9.61 (0.53)	*F* (38,957) = 19.69, *p* < 0.001, *η* ^2^ = 0.01
**Race‐ethnicity**					
Asian	3.26	1.97	0.27	0.70	*χ* ^2^(21) = 2867.60, *p* < 0.001, Cramer's *V* = 0.33
Biracial/multiracial	9.07	10.36	7.96	6.97	
Black	**2.85**	8.72	**33.08**	36.24	
Hispanic or Latino/a/x	**8.40**	21.74	**33.93**	19.51	
Native American /Alaskan/Hawaiian	0.11	0.42	0.58	0.70	
Other race‐ethnicity	0.27	0.58	0.45	1.05	
Pacific Islander	0.03	0.08	0.13	0	
White	**68.62**	48.55	**11.28**	13.24	
* Missing*	7.40	7.59	12.31	21.60	
**Country of origin**					
US‐born	88.85	88.90	85.26	75.61	*χ* ^2^(3) = 6.28, *p* = 0.10, Cramer's *V* = 0.03
Not US‐born	2.80	**3.60**	**2.43**	2.79	
* Missing*	7.35	12.31	12.31	21.6	
**Gender**					
Cisgender female	43.60	45.17	**44.04**	**30.31**	*χ* ^2^(9) = 15.48, *p* = 0.08, Cramer's *V* = 0.02
Cisgender male	48.98	47.11	**43.46**	**47.39**	
Transgender female	0.03	0.03	0.04	0	
Transgender male	0	0.03	0	0	
* Missing*	7.40	7.67	12.45	22.30	
**Family structure**					
Dual caregiver	**83.34**	68.21	**41.30**	34.84	*χ* ^2^(3) = 1201.10, *p* < 0.001, Cramer's *V* = 0.37
Single caregiver	**9.26**	24.01	**44.18**	41.46	
* Missing*	7.40	7.78	14.52	23.69	
*School‐based indicators of functioning*					
**GPA, age 11–12**	3.54 (0.45)	3.41 (0.58)	3.17 (0.71)	2.85 (0.81)	*F* (38,328) = 225.80, *p* < 0.001, *η* ^2^ = 0.08
**Drop in grades in the past year**					
Yes	**8.05**	15.54	**24.00**	19.61	*χ* ^2^(3) = 294.02, *p* < 0.001, Cramer's *V* = 0.17
No	**91.87**	84.35	**75.73**	80.84	
* Missing*	0.08	0.11	0.18	0	
**Detention or suspension in the past year**					
Yes	**3.36**	8.45	**18.47**	25.09	*χ* ^2^(3) = 466.19, *p* < 0.001, Cramer's *V* = 0.22
No	**96.50**	91.47	**81.35**	74.91	
* Missing*	0.13	0.08	0.18	0	
**Ever repeated a grade**					
Yes	**4.14**	6.04	12.90	**27.18**	*χ* ^2^(3) = 325.64, *p* < 0.001, Cramer's *V* = 0.18
No	95.78	93.91	86.92	72.82	
* Missing*	0.08	0.06	0.18	0	

*Note*: Total *N* = 9839, cognitive resilience *n* = 2225, cognitive vulnerability *n =* 287, average‐average *n =* 3611, advantaged *n* = 3716. All profiles differed across all demographic variables, except gender and country of origin, as determined by ANOVA and Chi‐square Goodness‐of‐Fit tests, and significance was assessed based on Bonferroni‐corrected *p* values (*α* = 0.008). Bold indicates cells that contributed the largest residual to the group differences in profile membership.

Abbreviations: GPA = grade point average, *M* = mean, SD = standard deviation.

Youth racial‐ethnic identity (*V* = 0.33) and family structure (*V* = 0.37) were most strongly associated with profile membership; there was a greater proportion of Black and Hispanic/Latinx youth and a lower proportion of White youth in the lower SER profiles (CV and CR), relative to the higher SER profiles (ADV and AVG), supporting our second hypotheses. Results from tests of measurement invariance provided mixed support for the generalizability of the profile structure. Evidence supported invariance for country of origin, partial invariance for gender, and non‐invariance for race‐ethnicity and family structure (see Table S8 for full model comparisons).

To understand the structural challenges and affordances that different groups of youth face, we also examined the associations between profile membership and youth GPA, grades dropping over the past year, past year detention or suspension, and repeated grade level at any point during schooling. Youth in both lower SER profiles were significantly more likely to receive a detention or suspension in the past year (*V* = 0.15), report a drop in grades (*V* = 0.12), and repeat a grade level (*V* = 0.13), relative to youth in profiles characterized by higher SER.

### 3.3 Aim 3: Ecological Risk and Protective Factors for Low‐SER Youths’ Cognitive Functioning

**FIGURE 4:**
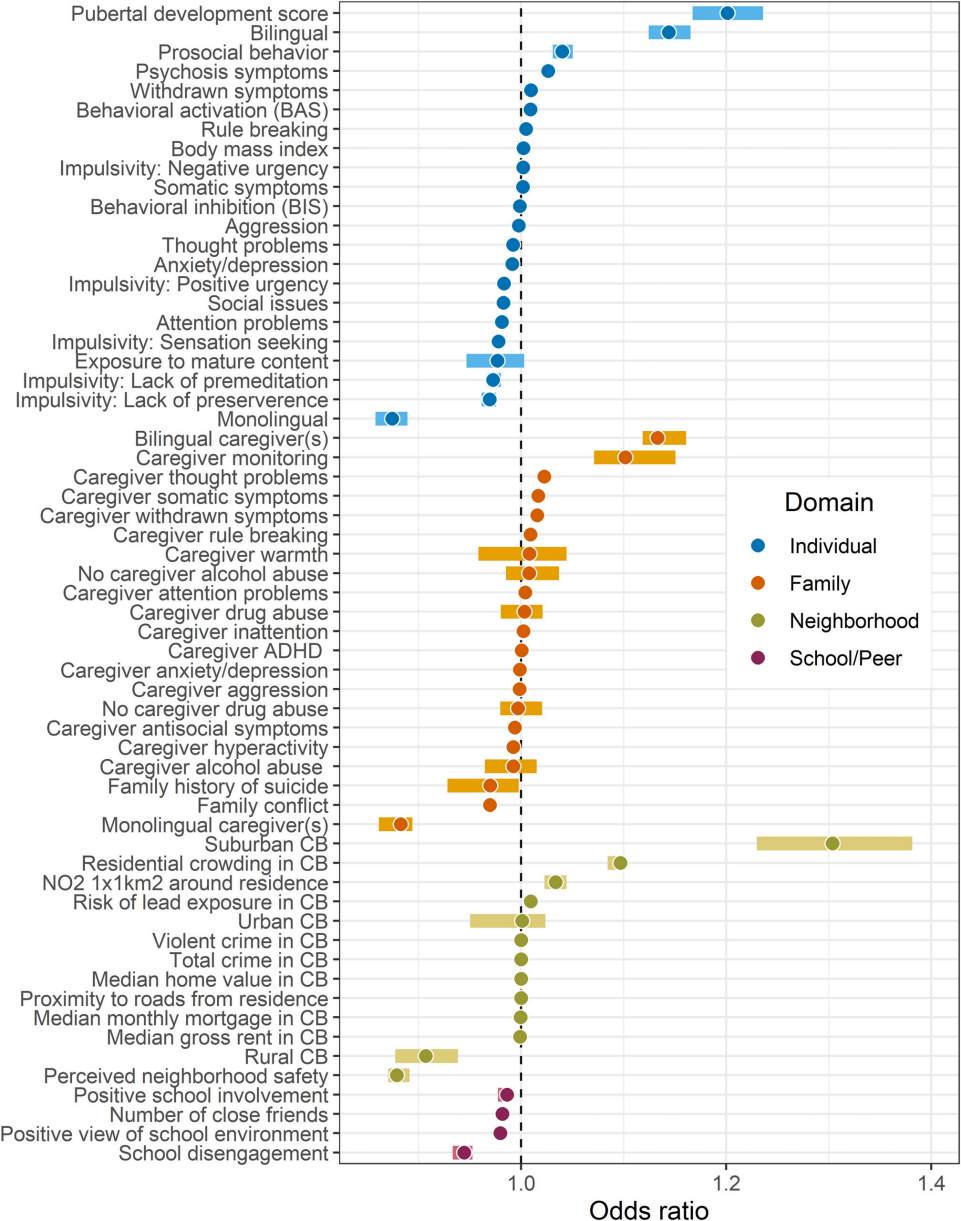
Ecological predictors of low‐SER youths’ odds of membership in the resilient profile over the vulnerable profile (controlling for age). *Note*: Total *N* = 9839. Odds ratios (OR) greater than one indicate that the predictor was associated with an increase in youths’ odds of being characterized as CR over CV, whereas ORs less than one indicate that the predictor was associated with a decrease in youths’ odds of being characterized as CR over CV. Categorical variables (e.g., urbanicity) were dummy coded. ORs are derived from bootstrapped coefficient estimates (*b* = 1000). Dark circles represent the median of the bootstrapped distribution, and error bars represent 95% confidence intervals. ADHD = attention‐deficit/hyperactivity disorder, CB = census block, NO_2_ = nitrogen dioxide.

Finally, we conducted multinomial logistic ridge regression to identify ecological predictors associated with profile membership. Here, we report predictors found to enhance socioeconomically disadvantaged youths’ odds of being classified into the profile with higher cognitive domain scores (i.e., CR) versus lower cognitive domain scores (i.e., CV; Figure 4), with results for all other pairwise comparisons available in Figures S2–S6. Moreover, we center our results on predictors that had meaningful effects, which we defined as a ≥5% change in odds (i.e., OR ≥ 1.05 or OR ≤ 0.95), after adjusting for age (see Table S9 for all OR estimates). Consistent with our third hypothesis, protective factors for low‐SER youth were distributed across ecological systems rather than concentrated within one ecological context.

Twenty‐three ecological factors significantly predicted *greater* odds of lower‐SER youth being classified as “resilient” versus “vulnerable.” Living in a suburban Census tract (OR = 1.30, 95% CI = 1.23‒1.38), a 1‐unit increase in pubertal development (OR = 1.20, 95% CI = 1.17‒1.24), being bilingual (OR = 1.14, 95% CI = 1.12‒1.17), having a bilingual caregiver (OR = 1.14, 95% CI = 1.12‒1.16), a 1‐unit increase in parental monitoring (OR = 1.10, 95% CI = 1.07‒1.15), and a 1‐unit increase in residential crowding (OR = 1.10, 95% CI = 1.08‒1.10) were each associated with a meaningful increase in odds of classification into the “resilient” profile. By contrast, twenty‐seven factors significantly predicted a *reduction* in odds of “resilient” profile membership versus “vulnerable,” including being monolingual (OR = 0.87, 95% CI = 0.86‒0.89), having a monolingual caregiver (OR = 0.88, 95% CI = 0.86‒0.89), a 1‐unit increase in neighborhood safety (OR = 0.88, 95% CI = 0.87‒0.89), living in a rural Census block (OR = 0.91, 95% CI = 0.88‒0.94), and a 1‐unit increase in school disengagement (OR = 0.94, 95% CI = 0.93‒0.95). Finally, eleven factors, including caregiver warmth, caregiver drug or alcohol abuse, caregiver ADHD behaviors, urban residence, BMI, negative urgency, exposure to mature media, and behavioral inhibition, did not significantly change youths’ odds of being in either profile (i.e., the 95% CI included 1).

## 4 Discussion

Using a person‐centered modeling approach and a large population‐based dataset of early adolescents in the United States, this study describes variability in patterns of cognitive performance and SER within the home and neighborhood contexts. Three profiles captured patterns typically identified in academic research, policy, and practice: “Advantaged” (high cognitive performance, high SER), “Average‐Average” (average cognitive performance, average SER), and “Cognitive Vulnerability” (low cognitive performance, low SER). A fourth profile, “Cognitive Resilience,” described youth who demonstrated average cognitive performance despite experiencing significant family socioeconomic disadvantage and the highest degree of neighborhood socioeconomic disadvantage. Notably, the number of low‐SER youth who demonstrated cognitive vulnerability (*n* = 287) was far smaller than the number who were characterized by resilience (*n* = 2225), challenging stereotypes about youth in low‐resourced settings and highlighting the need for strengths‐based research frameworks. Next, we identified factors within individuals and at the family‐, school‐, and neighborhood‐levels that increased low‐SER youths’ likelihood of demonstrating cognitive resilience. Consistent with multisystem resilience perspectives (Masten et al. 2021), resilience‐promoting factors were present at all levels of ecological influence. Importantly, this study adds to a growing body of work calling for strengths‐based perspectives in developmental science and investigations that probe functional heterogeneity within adversity‐exposed populations (Ellis et al. 2022). To support this aim, we used LPA to identify subgroups that reflect joint patterns of cognitive functioning and access to SER rather than modeling these domains in isolation.

### 4.1 Commonality of Cognitive Resilience

A large proportion of children in this sample demonstrated cognitive resilience to socioeconomic adversity (i.e., 88.6% of low‐SER youth). Although the “resilient” and “vulnerable” profiles differed in average family and neighborhood SER scores, both groups experienced substantial disadvantage. For example, the mean income‐to‐needs ratios were 1.16 for the “resilient” profile and 1.50 for the “vulnerable” profile, both falling below 185% of the federal poverty line and aligning with eligibility for public benefits such as free or reduced‐price lunch (USDA 2024). The relatively high cognitive performance of “resilient” youth mirrors findings from other adversity‐exposed populations. For instance, a study examining profiles of social, academic, occupational, and emotional competence in emancipated foster youth found that 48% of youth were broadly resilient, 16% were broadly maladapted, and the remaining youth were characterized by mixed outcomes (Yates and Grey 2012). Similar estimates of behavioral and mental health resilience have been identified with person‐centered studies of adolescents who are unhoused (e.g., 40% in Herbers et al. 2020). The current study contributes to this line of research by investigating the commonality of resilience regarding cognitive functioning, challenging deficit‐based models that understate low‐SER youths’ resilience.

At the same time, an examination of within‐profile heterogeneity in cognitive performance suggests that some skills may be more impacted by socioeconomic disadvantage during early adolescence than others (Ellis et al. 2022; Frankenhuis and de Weerth 2013). For youth in the “resilient” profile, their lowest scores were, on average, in the VR domain, whereas their scores in the SF and memory domains were within 0.5 SD of the mean scores demonstrated by youth in the high‐SER profiles. That “resilient” youths’ SF scores were relatively unaffected is consistent with prior work testing specific skills within this domain, such as cognitive flexibility and attention shifting, which has found evidence of enhancements among adversity‐exposed youth (e.g., Young et al. 2022). That said, we caution that our cross‐sectional design and characterization of profiles using cognitive domain scores, rather than individual task scores, limit our ability to make inferences about adaptive processes within specific cognitive abilities. Instead, we interpret this pattern as evidence of domain‐specific resilience in SF and memory domains, consistent with multisystem resilience theory (Masten et al. 2021) and recent work finding associations between SER and task‐general cognitive outcomes (e.g., Bignardi et al. 2024; Vermeent et al. 2024). To complement these findings, future research using longitudinal data on adversity exposure may apply person‐centered methods at the task level to test adaptation‐based hypotheses.

“Resilient” youth being more negatively impacted within the VR domain is also consistent with well‐documented findings of impairments in reading and language as a function of socioeconomic disadvantage (e.g., Calvo and Bialystok 2014). It is important to note, however, that the VR latent factor that we estimated in this sample also included performance on a task assessing working memory updating, which has also been shown to remain unaffected or be strengthened among youth exposed to harsh and unpredictable environments (Young et al. 2022). However, adaptive patterns for working memory updating are limited to studies using ecologically relevant stimuli (e.g., faces, places) rather than the abstract stimuli featured in the assessment administered in the ABCD Study. This could explain both the factor structure of cognition that we identified and why “resilient” youth were more impacted in this domain, in contrast to previous work, highlighting the need to understand the role of measurement and task design in documenting patterns of cognitive risk and resilience to adversity.

Despite heterogeneity in cognitive performance among low‐SER youth, our examination of the sociodemographic characteristics of the four profiles reinforced well‐documented racial‐, ethnic‐, and class‐based disparities. The overrepresentation of Black and Hispanic/Latinx youth in the socioeconomically disadvantaged profiles (i.e., CR and CV) reflects a history of systemic economic disenfranchisement (Massey 1990) and constrained access to educational opportunities (Merolla and Jackson 2019) in racial‐ethnic minoritized communities within the United States. Moreover, “resilient” and “vulnerable” youth were far more likely to receive school‐based disciplinary action than “advantaged” and “average” youth, consistent with a previous report in this sample (Fadus et al. 2021). Considering the disproportionate rate of disciplinary action faced by Black, Hispanic/Latinx, and Native American students (Riddle and Sinclair 2019), these results implicate the intersectional race‐ and class‐based subjugation of children and adolescents.

### 4.2 Risk and Protective Factors for Cognitive Development

We also examined the relative influence of 55 ecological factors in predicting socioeconomically disadvantaged youths’ odds of membership in profiles characterized by resilience versus vulnerability, while controlling for multicollinearity through penalized ridge regression. The most powerful individual‐level predictor of low‐SER youths’ odds of demonstrating cognitive resilience versus vulnerability was pubertal timing (controlling for chronological age), wherein earlier‐developing youth were much more likely to be classified as CR than CV. Hypotheses rooted in Life History Theory suggest that early puberty may be a tradeoff intended to prioritize reproductive success and fitness in adverse contexts (Ellis 2004). Although early puberty is linked to negative health consequences, including internalizing and externalizing psychopathology (Ullsperger and Nikolas 2017), Barton et al. (2023) showed that, for racial‐ethnic minoritized youth living in socioeconomically disadvantaged neighborhoods, accelerated puberty was associated with advanced EF skills. Given that accelerated pubertal maturation has been observed in tandem with accelerated trajectories of brain development (Holm et al. 2023), the advanced pubertal status of CR youth in this sample may reflect experience‐dependent adaptations in neurocognitive brain structures (Ellis et al. 2022).

Bilingualism was the second most impactful individual‐level predictor of resilience for socioeconomically disadvantaged youth. Nearly forty percent of the sample was bilingual, with Spanish as the most spoken secondary language (70.5%; Table S10). Our results align with prior research demonstrating that class‐based cognitive disparities are narrower in bilingual compared to monolingual populations (e.g., Engel de Abreu et al. 2012). Bilingualism is linked to greater benefits in cognitive flexibility and selective attention relative to other components of EF (Ware et al. 2020), consistent with our findings that CR youth were relatively unaffected in the SF domain. Moreover, studies in younger low‐SER youth have found that such benefits may be coupled with disadvantages on tests of verbal ability (e.g., Calvo and Bialystok 2014), paralleling the results presented here. Given that lower SER is associated with reduced access to cognitive stimulation (see Hyde et al. 2020 for a review), bilingual youths’ repeated engagement of brain networks dually involved in language switching and broader EF capabilities (e.g., task switching) may serve as an important source of cognitive stimulation in socioeconomically disadvantaged contexts (Coderre et al. 2016). As having a bilingual caregiver was also a significant predictor of resilience in socioeconomically disadvantaged youth, future research should examine whether these protective effects are confounded by family‐based cultural capital, such as ethnic‐racial socialization mediated through caregiver‐youth communication (Yosso 2005). It could also be argued that these results are evidence for an “immigrant paradox,” wherein those who immigrate might be healthier or have more pre‐existing resources (Marks et al. 2014); however, the results here show that “vulnerable” youth were more likely to be born outside of the United States than “resilient” youth, challenging this narrative.

Beyond caregiver bilingualism, a major family‐level factor that predicted resilience was greater caregiver monitoring. This finding adds to the long‐established protective effect of monitoring on mental health and academic resilience and the prevention of risk‐taking behaviors, especially for youth living in unsafe or under‐resourced neighborhoods (Ozer et al. 2017). A recent paper with participants in the ABCD Study identified that a greater degree of caregiver monitoring, but not warmth, mediated the association between youths’ household income and general cognitive ability scores (Keller et al. 2023). Given that CR youth were exposed to significantly greater levels of neighborhood disadvantage and felt more unsafe in their neighborhoods than CV youth, a greater level of caregiver monitoring may reflect differences in neighborhood violence and crime that necessitate a greater level of supervision to lessen the risk of harm (Ozer et al. 2017). At the same time, caregivers with lower SER are disproportionately more likely to work inflexible and non‐traditional work hours that limit opportunities for monitoring (Enchautegui et al. 2015), highlighting the need for further investigation into how barriers to caregiver monitoring may exacerbate class‐based disparities in youth development.

Of the risk and protective factors in the more distal school and neighborhood domains, urbanicity had the largest impact on youths’ classification as resilient or vulnerable. Living in a suburban area promoted cognitive resilience, while living in a rural area was associated with cognitive vulnerability; profile membership was not significantly associated with living in an urban area. It is not surprising that rurality lessened youths’ odds of cognitive resilience in our sample, given the well‐documented disparities in social safety net resources for low‐income families across the urban‐rural continuum (Shapiro 2021). For example, compared to youth living in rural areas, suburban youth are more likely to attend schools with greater per‐pupil expenditures (Kettler et al. 2015) and live closer to built‐environment features linked to positive academic and health outcomes (Johnson and Johnson 2015; Roscigno et al. 2006). At the same time, suburban neighborhoods may have advantages that go beyond proximity to resources to include cultural and community assets such as neighborhood‐level racial‐ethnic diversity and access to neighborhood peer interactions (Parker et al. 2018; Witherspoon et al. 2023).

Within the school domain, school disengagement was associated with the greatest reduction in low‐SER youths’ odds of demonstrating cognitive resilience. Surprisingly, membership in the “vulnerable” profile was also associated with more positive perceptions about the school environment and opportunities for student involvement. Thus, despite viewing their schools as being safe and supportive, “vulnerable” youth may also have maintained a heightened sense of school disengagement. Additionally, a greater proportion of “vulnerable” youth received detention or were suspended in the past year, so our results may reflect bidirectional pathways in which school disengagement begets involvement in disciplinary action and vice versa (Pyne 2019). Further research is needed to elucidate how disengagement, a key predictor of grades, behavioral problems, and high school dropout (Henry et al. 2012), can be targeted to promote cognitive resilience in low SER contexts.

Lastly, it is important to contextualize some of the counterintuitive predictors of resilience, including that greater exposure to higher concentrations of NO_2_, greater levels of residential crowding, and lower youth perceptions of neighborhood safety were each associated with membership in the “resilient” versus the “vulnerable” profile. These factors are well‐documented risks to cognitive development (Evans 2021). It may be that “resilient” youths’ greater exposure to neighborhood risk factors reflects the fact that they had significantly less neighborhood SER than “vulnerable” youth. Given the greater proportion of “vulnerable” youth living in rural areas compared to “resilient” youth, it may also be that natural variation in exposure to air pollution stands as a confounder or statistical artifact.

### 4.3 Limitations

Despite the novelty of applying person‐centered and data‐driven approaches to characterizing heterogeneity in cognitive performance within socioeconomically disadvantaged youth, this study is not without limitations. First, our characterization of youth as “resilient” or “vulnerable” was based on cross‐sectional data. Many definitions of resilience emphasize its nature as a dynamic process wherein resilient youth continue to acquire knowledge and skills in the process of adapting to their ever‐changing environments (e.g., Masten et al. 2021). Researcher‐imposed labels of resilience may not reflect youths’ perceptions of their own cognitive functioning and obscure strengths and difficulties in other areas of functioning (e.g., mental and physical health). Additionally, despite using random starts to minimize the risk of local maxima in LPA, we did not conduct bootstrapped or split‐sample replications. Future research should test for measurement invariance at later time points, evaluate resilience in multiple domains, and further assess the stability of these latent profiles. Second, the neurocognitive battery did not assess specific cognitive abilities that may be enhanced among people living in poverty (i.e., procedural memory; Dang et al. 2016; Leonard et al. 2015), limiting our ability to detect resilience in other areas of cognition. Third, the current study cannot speak to causal predictors of cognitive resilience; future research should employ experimental designs using longitudinal data. Fourth, although the ABCD Study utilized probability sampling of US schools to minimize selection bias, the demographics of the baseline sample deviate from the population of same‐aged youths in regards to caregiver education, family structure, and income (Gard et al. 2023), limiting the generalizability of our results. Finally, many neighborhood‐level predictors of profile membership relied on zip code level estimates, but studies have shown that there is substantial variation in exposures within zip codes (Browning et al., 2021); further investigation using more spatially localized data is needed.

## 5 Conclusions

Improving our understanding of the supports and barriers for cognitive development in the context of socioeconomic disadvantage will require person‐centered analyses, multi‐domain assessments of resilience, and longitudinal designs. As demonstrated in this study, deficit‐based stereotypes about lower‐SER youth may not generalize, as a large proportion of lower‐SER youth in this population‐based nationwide sample performed on par with their higher‐SER peers. Future research should use methods that allow us to highlight the duality of strengths and weaknesses as well as youths’ adaptive capacity across multiple domains of development. Ultimately, research must move toward capturing the diversity of lived experiences among socioeconomically disadvantaged youth, including access to supports in multiple ecological contexts.

## Funding

The authors have nothing to report.

## Ethics Statement

The ABCD Study protocol was approved by each study site's Institutional Review Board. Data access from authors was deemed “exempt” by the [BLINDED FOR REVIEW] Institutional Review Board (IRB#2240843‐1)

## Conflicts of Interest

The authors declare no conflicts of interest.

## Supporting information




**Supporting File**: desc70105‐sup‐0001‐SuppMat.docx

## Data Availability

The data and materials necessary to reproduce the analyses presented here are publicly accessible through the ABCD Study. The data and materials used in this report can be accessed at the following URL: https://doi.org/10.15154/1523041. The analytic code necessary to reproduce the analyses presented in this paper is publicly accessible. Code is available at the following URL: https://osf.io/j3rvs/. The analyses presented here were not preregistered. Data used in the preparation of this article were obtained from the Adolescent Brain Cognitive Development (ABCD) Study (https://abcdstudy.org), held in the NIMH Data Archive (NDA).

## Endnotes

1 We use the term *socioeconomic resources (SER)* in place of *socioeconomic status (SES)* to reflect a multidimensional conceptualization of family and neighborhood conditions, capturing both material and contextual assets that influence child development. We favor this terminology to emphasize resource‐based inputs rather than hierarchical or status‐based labels. However, we acknowledge that this choice may reduce comparability across studies relying on conventional SES terminology.
