# Supplementary material for: Cognitive Resilience and Vulnerability to Socioeconomic Disadvantage: Predictors Across Individual, Family, School, and Neighborhood Contexts
Source: Dev Sci. 2025 Dec 11;29(1):e70105. doi: 10.1111/desc.70105 (PMC12696620; doi:10.1111/desc.70105)
Supplement: Supplementary file 1 — Supporting File: desc70105‐sup‐0001‐SuppMat.docx [file DESC-29-e70105-s001.docx]

**Cognitive resilience and vulnerability to socioeconomic disadvantage: Predictors across individual, family, school, and neighborhood contexts**

**Supplementary Materials**

# Supplementary Methods

**Eligibility criteria for the Adolescent Brain and Cognitive Development (ABCD) Study.**

Youth who were not English language proficient, with severe sensory, intellectual, medical, or neurological issues, or with MRI contraindications were ineligible to participate (Garavan et al., 2018).

**Description of cognitive assessments.**

*Picture Vocabulary***.** Used to measure verbal language ability, the NIH Toolbox (NIHTB) Picture Vocabulary task asks participants to match a word that is presented audibly to one of four pictures (Gershon et al., 2014; Weintraub et al., 2013). The task is approximately 3 minutes in length. For youth with valid data, M = 84.54 (SD = 8.12; Min = 47; Max = 119).

*Oral Reading Recognition Task*. Used to measure reading skills, the NIHTB Oral Reading Recognition Task asks participants to verbally read letters and words that are visually presented (Weintraub et al., 2013). The task is approximately 4 minutes in length. For youth with valid data, M = 90.96 (SD = 6.94; Min = 59; Max = 119).

*List Sorting Working Memory Test*. Used to measure working memory ability, the NIHTB List Sorting Working Memory Test asks participants to remember the sequence of a quickly-presented list of stimuli and then recall the stimuli in a newly defined criterion (e.g., largest to smallest; Weintraub et al., 2013). The task is approximately 7 minutes in length. For youth with valid data, M = 96.73 (SD = 12.13; Min = 36; Max = 136).

*Wechsler Intelligence Test for Children-V (WISC-V) Matrix Reasoning subtest*. Used to measure nonverbal abstract reasoning and fluid intelligence, the Matrix Reasoning subtest asks participants to view an incomplete series of stimuli and select one of four possible options that complete the sequence (Wechsler, 2014). The task takes up to 10 minutes to complete. For youth with valid data, M = 17.89 (SD = 3.84; Min = 0; Max = 32).

*Pattern Comparison Processing Speed Test*. Used to measure visual information processing, the NIHTB Pattern Comparison Processing Speed Test asks participants to identify whether two visually presented stimuli are identical or not identical as quickly as possible (Carlozzi et al., 2015; Weintraub et al., 2013). The task is approximately 4 minutes in length. For youth with valid data, M = 87.90 (SD = 14.59; Min = 35; Max = 140).

*Flanker Task*. Used to measure inhibitory control, the NIHTB Flanker Task asks participants to attend to the specific orientation of a stimulus and respond accordingly, while inhibiting distractor stimuli (Eriksen & Eriksen, 1974; Weintraub et al., 2013). The task is approximately 3 minutes in length. For youth with valid data, M = 94.00 (SD = 9.18; Min = 53; Max = 116).

*Dimensional Change Card Sort Task.* Used to measure cognitive flexibility, the NIHTB Dimensional Change Card Sort Task asks participants to select matching pairs based on a shared characteristic (e.g., same color) as quickly and accurately as possible (Weintraub et al., 2013; Zelazo et al., 2014). The characteristic that youth should attend to changes often and is signaled by an audible cue. The task is approximately 4 minutes in length. For youth with valid data, M = 92.48 (SD = 9.50; Min = 50; Max = 120).

*Picture Sequence Memory Test*. Used to measure episodic memory, the NIHTB Picture Sequence Memory Test asks participants to remember a list of events and recall them in the correct sequence (Bauer et al., 2013; Dikmen et al., 2014; Weintraub et al., 2013). The task is approximately 7 minutes in length. For youth with valid data, M = 102.80 (SD = 12.10; Min = 76; Max = 136).

*Rey Auditory Verbal Learning Test (RAVLT)*. Used to measure short- and long-term memory retention and recall, the Rey Auditory Verbal Learning Test asks participants to listen and recall a list of fifteen words over the course of five learning trials (Schmidt, 1996). Afterwards, participants are presented with a distractor list of words. To assess short-term memory, the experimenters counted the number of words from the original list that the participant could recall immediately following the distractor trial. Long-term memory retention of the original list was assessed in the same manner, after a 30-minute delay period. The task is approximately 5 minutes in length. For youth with valid short-term memory data, M = 9.65 (SD = 3.06; Min = 0; Max = 15). For youth with valid long-term memory data, M = 9.18 (SD = 3.23; Min = 0; Max = 15).

**Description of socioeconomic resource (SER) indicators.**

*Neighborhood Socioeconomic Disadvantage index.* Used to measure the socioeconomic assets of youths’ neighborhoods, this index was constructed by averaging nine standardized variables measured at the census tract-level and derived via geocoding of participant’s primary home address (Fan et al., 2021; Taylor et al., 2020). For youth with valid data, *M* = 0.02 (SD = 0.85; Min = -1.49; Max = 3.92). The nine variables include (R = reverse scored):

1. Percentage of population below 138% of the poverty threshold
2. Percentage of population aged ≥ 25 y with at least a high school diploma (R)
3. Percentage of owner-occupied housing units (R)
4. Income disparity defined by Singh as the log of 100 x ratio of the number of households with < $10,000 annual income to the number of households with > $50,000 annual income
5. Median family income (R)
6. Percentage of occupied housing units without a motor vehicle
7. Percentage of families below the federal poverty level
8. Percentage of single parent households
9. Percent of labor force population aged ≥ 16 y that is unemployed

In the CFA of SER, the index was standardized via z-score transformation (i.e., *M* = 0, SD = 1), multiplied by -1 so that a more positive score corresponded to a *lower* level of neighborhood disadvantage.

*Child Opportunity Index (COI): Health and Environment subscale.* The COI is used to measure neighborhood resources and traits associated with the promotion of children’s health, cognitive, and socioemotional development. This score was constructed using census tract-level data collected in 2015. See <https://www.diversitydatakids.org/research-library/research-brief/how-we-built-it> for technical documentation. COI subscale scores correspond with their neighborhood’s percentile rank. The Health and Environment subscale assesses the health (e.g., access to green space) and environmental well-being (e.g., ozone concentration) of a participant’s neighborhood, compared to all other neighborhoods in the U.S. For youth with valid data, *M* = 57.55 (SD = 30.32; Min = 1; Max = 100).

*COI: Education subscale.* The COI is used to measure neighborhood resources and traits associated with the promotion of children’s health, cognitive, and socioemotional development. This score was constructed using census tract-level data collected in 2015. See <https://www.diversitydatakids.org/research-library/research-brief/how-we-built-it> for technical documentation. COI subscale scores correspond with their neighborhood’s percentile rank. The Education subscale assesses educational resources (e.g., teacher experience) and academic quality indicators (e.g., third grade reading proficiency) of a participant’s neighborhood, compared to all other neighborhoods in the U.S. For youth with valid data, M = 60.00 (SD = 29.99; Min = 1; Max = 100).

*COI: Social and Economic subscale.* The COI is used to measure neighborhood resources and traits associated with the promotion of children’s health, cognitive, and socioemotional development. This score was constructed using census tract-level data collected in 2015. See <https://www.diversitydatakids.org/research-library/research-brief/how-we-built-it> for technical documentation. COI subscale scores correspond with their neighborhood’s percentile rank. The Social and Economic subscale assesses economic opportunities (e.g., employment rate) and socioeconomic resources (e.g., public assistance rate) of a participant’s neighborhood, compared to all other neighborhoods in the U.S. For youth with valid data, M = 58.27 (SD = 30.92; Min = 1; Max = 100).

*Material Hardship.* Used to measure perceptions of financial hardship experiences, caregivers were asked to indicate whether or not their family experienced seven types of financial hardships over the past 12 months. For example, the items included “Didn't pay the full amount of the rent or mortgage because you could not afford it?” and “Had services turned off by the gas or electric company, or the oil company wouldn't deliver oil because payments were not made?”. The material hardship score was calculated by taking the sum of items endorsed. For youth with valid data, M = 0.48 (SD = 1.10; Min = 0; Max = 7). In the CFA of SER, this variable was standardized via z-score transformation (i.e., M = 0, SD = 1), multiplied by -1 so that a more positive score corresponded to a *lower level* of material hardship.

*Caregiver Education.* Used to measure the educational attainment of youths’ caregivers, caregivers indicated the highest level of education that they and (if applicable) the participant’s second caregiver have completed. The score was calculated by first converting educational categories to years of education (e.g., “2^nd^ grade” became 3 years, “Bachelor’s degree” became 17 years) and using the score for the caregiver with the highest level of education. For youth with valid data, M = 17.07 (SD = 2.67; Min = 3; Max = 21).

*Income-to-needs (INR) Ratio.* Used to measure household income while taking family size into account, the INR used the caregiver-reported data, including the income category that encompassed their combined household income (e.g., “$35,000 through $49,999”) and the number of individuals currently living in the household. To get the INR, we divided the median of the selected household income category was divided by the 2017 US federal poverty line for their family size (e.g., $24,600 for a family of 4 per the US Census Bureau’s 2017 guidelines). For youth with valid data, M = 3.74 (SD = 2.50; Min 0.03; Max = 12.44).

**Validation of the 3-class cognition factor structure.**

The three-factor structure of youth cognitive performance at baseline was validated by correlating the extracted factor scores at baseline with individual task scores from the 2-year follow up data collection (i.e., when youth were 11-12 years of age). One indicator from each factor (i.e., Speed-Flexibility; Memory; Verbal-Reasoning [VR]) was randomly selected to be the 2-year validation variable. As expected, the 2-year follow up indicators were most highly correlated with the hypothesized baseline factor score (e.g., NIHTB Oral Reading Recognition Task scores at the 2-year follow up were most strongly correlated with VR factor scores at baseline; r = 0.70). All correlations were statistically significant at *p* < 0.001. See Figure S1.

There are slight differences between our factor structure of cognition and the structure identified by Sripada et al., 2021. First, given the focus on examining brain function, youth who were missing usable neuroimaging data were excluded from Sripada et al. (2021) (final *N* = 5,937), whereas our study did not take neuroimaging exclusion criteria into account (final *N* = 9,839). Second, we excluded scores from the Little Man Task because the factor loading was not greater than the recommended value of 0.3 (Guadagnoli & Velicer, 1988). Third, because we were interested in examining youths’ relative function across specific domains of cognition, we elected not to use the domain-general cognitive bifactor term that had been originally included in the model used by Sripada. Fourth, there were some differences in our naming of the latent factors (i.e., “Visual/Spatial” became “Verbal and Reasoning”, “Speed/Flexibility” became “Speed and Flexibility”, and “Learning/Memory” became “Memory”). These changes were intended to better represent the overlapping cognitive functions tested in the tasks encompassing each factor.

**Identification and validation of the 4-class latent profile model.**

We justified our decision to use the 4-class latent model over the 5-class latent model by evaluating model fit statistics (AIC/BIC, log likelihood), indicators of profile classification (entropy, posterior probabilities), and the conceptual interpretability of each profile (i.e., our ability to distinguish each profile as describing a unique pattern of cognitive scores and SER). Although the addition of a fifth profile improved the log likelihood, BIC, and the AIC, the entropy value depreciated below recommended values (Table S4; Table S5), and the pattern of SER and cognitive factor scores for youth in this profile did not meaningfully deviate from youth in the existing 4-profile solution. Specifically, two profiles in the 5-profile model were characterized as having the highest levels of family and neighborhood SER relative to the other classes, as well as very similar standardized means for cognitive domain scores (VR, SF, and MEM). The average latent class posterior probability was suboptimal for one of these profiles (0.66), indicating high conceptual overlap between two profiles in the 5-profile solution. Together, these empirical and theoretical considerations informed our selection of the 4-profile model as the best-fitting and most parsimonious solution.

Moreover, the structure of the 4-profile solution was validated using two methods. First, we examined profile-level means for the only nationally normed cognitive assessment included in the ABCD study’s baseline neurocognitive battery, scaled scores on the Matrix Reasoning subtest of the WISC-V. For this subtest, the “Average” range is defined as a score between 8 to 12 (Wechsler et al., 2014). Aside from CV, whose mean score fell into the “Low Average” range (i.e., 6 to 7), all other profiles means were characterized as “Average”. All profiles significantly differed on WISC-V Matric Reasoning scores (*p* < 0.001), and the effect of profile membership was moderate (η^2^ = 0.08).

Second, we validated profiles using caregiver-reported class grades at the 2-year ABCD Study follow-up (i.e., when youth were on average 11.99 years old [SD = 0.66]) and calculating youths’ grade point average (GPA). GPA was chosen as the second validating variable for its consistently documented association with cognitive test scores (Roth et al., 2015) and SER (Selvitopu & Kaya, 2023). According to ANOVA tests, GPA significantly differed between profiles (*p* < 0.001) and the association between profile membership and GPA was moderate-to-large in effect (η^2^ = 0.13). The ranking of profile-level average cognitive domain factor scores aligned with the relative ranking of GPAs (e.g., the profile with the lowest average cognitive domain factor scores, CV, had the lowest average GPA). Together, these results confirmed our characterization of the profiles as reflecting SER-dependent cognitive patterns that correspond with real-world metrics. See Table S6 for a comparison of average task scores across profiles.

# Supplementary Tables

## Table S1.

*Demographic differences between the analytic sample and the full ABCD Study sample.*

|  |  | Analytic sample  *N* = 9,839 | Full sample  *N* = 11,875 | Test statistic |
| --- | --- | --- | --- | --- |
|  |  | *%* | *%* | *Chi-sq (Cramer’s V)* |
| **Race-Ethnicity** | | | | 6.26 (0.02) |
|  | Asian | 2.03 | 1.86 |  |
|  | Biracial/Multiracial | 9.23 | 9.32 |  |
|  | Black | 12.82 | 12.59 |  |
|  | Hispanic or Latino/a | 19.39 | 18.41 |  |
|  | Native American/Alaskan/Hawaiian | 0.35 | 0.31 |  |
|  | Other Race | 0.45 | 0.43 |  |
|  | Pacific Islander | 0.07 | 0.10 |  |
|  | White | 46.67 | 47.87 |  |
|  | *Missing* | 8.99 | 7.46 |  |
| **Country of birth** | | | | 1.60 (0.01) |
|  | US-born | 88.05 | 88.24 |  |
|  | Not US-born | 3.01 | 2.73 |  |
|  | *Missing* | 8.94 |  |  |
| **Gender** | | | | 0.34 (<0.01) |
|  | Female | 43.89 | 44.01 |  |
|  | Male | 47.00 | 46.77 |  |
|  | Transgender Female | 0.03 | 0.03 |  |
|  | Transgender Male | 0.01 | 0.02 |  |
|  | *Missing* | 9.08 | 7.49 |  |
| **Family structure** | | | | 0.84 (0.01) |
|  | Dual caregiver | 66.87 | 68.48 |  |
|  | Single caregiver | 23.51 | 23.34 |  |
|  | *Missing* | 9.62 | 8.18 |  |
|  |  | *M (SD)* | *M (SD)* | *F (*η) |
| **Age (years)** | | 9.90 (0.62) | 9.92 (0.62) | 2.94 (<0.01) |

*Note.* The analytic sample was determined by removing youth missing baseline data on all cognitive assessments (n = 13), missing baseline data for all socioeconomic resource indicators (*n* = 4), and a randomly selected participant per family in cases where there were two participants from the same household (*n* = 2,019). The full and analytic samples did not statistically differ on any of the demographic variables (i.e., all *p* > 0.05).

## Table S2.

*Coding procedure for categorical sociodemographic and school-based functioning variables.*

| **Variable Name and Question Text** | **Variable Label** | **Coding Procedure** |
| --- | --- | --- |
| **Survey: *pdem02*** | | |
| Race-Ethnicity  (“What race do you consider the child to be?”; “Do you consider the child Hispanic/Latino/ Latina?”) | Asian | demo_race_a_p___18 (“Asian Indian”) *OR* demo_race_a_p___19 (“Chinese”) *OR* demo_race_a_p___20 (“Filipino”) *OR* demo_race_a_p___21 (“Japanese”) *OR* demo_race_a_p___22 (“Korean”) *OR* demo_race_a_p___23 (“Vietnamese”) *OR* demo_race_a_p___24 (“Other Asian”) == “Yes”  *AND* demo_ethn_v2 *==* “No” |
|  | Biracial/Multiracial | *Selected* “Yes” *for two or more racial categories (demo_race_a_p___[10 - 25])*  *AND* demo_ethn_v2 *==* “No” |
|  | Black | demo_race_a_p___11 (“Black/African American”) == “Yes”  *AND* demo_ethn_v2 *==* “No” |
|  | Hispanic or Latino/a | demo_ethn_v2 *==* “Yes” |
|  | Native American/ Alaskan/Hawaiian | demo_race_a_p___12 (“American Indian, Native American”) *OR* demo_race_a_p___13 (“Alaska Native”) *OR* demo_race_a_p___14 (“Native Hawaiian”) == “Yes”  *AND* demo_ethn_v2 *==* “No” |
|  | Other Race | demo_race_a_p___25 (“Other Race”) == “Yes”  *AND* demo_ethn_v2 *==* “No” |
|  | Pacific Islander | demo_race_a_p___15 (“Guamanian”) *OR*  demo_race_a_p___16 (“Samoan”) *OR*  demo_race_a_p___17 (“Other Pacific Islander”) == “Yes”  *AND* demo_ethn_v2 *==* “No” |
|  | White | demo_race_a_p___10 (“White”) == “Yes”  *AND* demo_ethn_v2 *==* “No” |
| Country of Origin  (“In which country was the child born?”) | US-born | demo_origin_v2 == "USA (United States of America - including territories such as Puerto Rico)” |
|  | Not US-born | demo_origin_v2 == *any other country (e.g., “131 = Pakistan”)* |
| Gender  (“What is the child's current gender identity?”) | Cisgender female | demo_gender_id_v2 == “Female” |
|  | Cisgender male | demo_gender_id_v2 == “Male” |
|  | Transgender female | demo_gender_id_v2 == “Transgender Female” |
|  | Transgender male | demo_gender_id_v2 == “Transgender Male” |
| Family Structure  (“Are you now married, widowed, divorced, separated, never married or living with a partner?”) | Dual caregiver | demo_prnt_marital_v2 == “Married” *OR* “Living with partner” |
|  | Single caregiver | demo_prnt_marital_v2 == “Widowed” *OR* “Divorced” *OR* “Separated” *OR* “Never married” |
| **Survey: *abcd_yksad01*** | | |
| Drop in grades in the past year  (“Has there been a drop in your grades in the past year?”) | Yes | kbi_y_drop_in_grades == “Yes” |
|  | No | kbi_y_drop_in_grades == “No” |
| Detention or suspension in the past year  (“In the past year, have you had any detentions or suspensions?”) | Yes | kbi_y_det_susp == “Yes” |
|  | No | kbi_y_det_susp == “No” |
| Ever repeated a grade  (“Have you ever repeated a grade?”) | Yes | kbi_y_grade_repeat == “Yes” |
|  | No | kbi_y_grade_repeat == “No” |

*Note.* All categorical sociodemographic variables (*race-ethnicity*, *country of origin*, *gender*, and *family structure*) were constructed using data from the ABCD Parent Demographics Survey (*pdem02*), completed by the caregiver participating in the study at baseline. The categorical school-based indicators of functioning (*drop in grades in the past year*, *detention or suspension in the past year*, and *ever repeated a grade*) were drawn from the ABCD Youth Diagnostic Interview for DSM-5 (Background Items; *abcd_yksad01*), which was completed by the youth participant.

## Table S3.

*Risk and promotive factors used to predict profile membership.*

| **Data source** | **Reporter** | **Predictor** | **Mean (SD) [Min-Max] or *N*** | **Sample item** |
| --- | --- | --- | --- | --- |
| ***Ecological Domain: Individual*** *(n = 22 predictors)* | | | | |
| Pubertal Development Scale | Youth and Caregiver (averaged between reporters) | Puberty score | 1.64 (0.42) [1 - 4] | *Youth*: "Have you noticed any skin changes, especially pimples?"  *Caregiver*: "Have you noticed any changes to their skin, especially pimples?" |
| Youth Acculturation Survey (Modified from PhenX) | Youth | Youth bilingualism | Bilingual:  *N* = 3,805  Monolingual:  *N* = 5,965 | "Besides English, do you speak or understand another language or dialect?" |
| Prosocial Behavior Survey | Caregiver | Prosocial behavior | 5.25 (1.22) [0 - 6] | "My child is… Helpful if someone is hurt, upset, or feeling ill." |
| Prodromal Psychosis Scale | Youth | Psychosis symptoms | 2.58 (3.51) [0 - 20] | "Did you feel that someone else, who is not you, has taken control over the private, personal, thoughts or ideas inside your head?" |
| Demographics | Caregiver | Age (months) | 118.80 (7.42) [107 - 133] | N/A |
| Behavioral Inhibition/ Behavioral Approach System Scales (BIS/BAS) | Youth | Behavioral approach | 20.97 (6.88) [0 - 39] | "When I see an opportunity to get something I want, I go for it right away." |
|  |  | Behavioral inhibition | 9.53 (3.76) [0 - 21] | "I am hurt when people scold me or tell me that I do something wrong." |
| UPPS-P Impulsive Behavior Scale | Youth | Lack of perseverance | 7.05 (2.27) [4 - 16] | "I almost always finish projects that I start." |
|  |  | Lack of premeditation | 7.73 (2.38) [4 - 16] | "I try to take a careful approach to things." |
|  |  | Negative urgency | 8.48 (2.65) [4 - 16] | "When I am upset I often act without thinking." |
|  |  | Positive urgency | 7.97 (2.97) [4 - 16] | "When I get really happy about something, I tend to do things that can lead to trouble." |
|  |  | Sensation seeking | 9.79 (2.69) [4 - 16] | "I like new, thrilling things, even if they are a little scary." |
| Child Behavior Checklist (CBCL) T-Scores | Caregiver | Youth aggressive behavior | 52.85 (5.50) [50 - 100] | "Physically attacks people" |
|  |  | Youth anxious/ depressed | 53.58 (6.05) [50 - 100] | "Feels worthless or inferior" |
|  |  | Youth attention problems | 54.02 (6.20) [50 - 97] | "Daydreams or gets lost in their thoughts" |
|  |  | Youth rule-breaking behavior | 52.81 (4.91) [50 - 84] | "Lying or cheating" |
|  |  | Youth social problems | 52.84 (4.76) [50 - 90] | "Not liked by other kids" |
|  |  | Youth somatic complaints | 55.05 (6.14) [50 - 88] | "Nausea, feels sick" |
|  |  | Youth thought problems | 53.93 (5.97) [50 - 84] | "Strange ideas" |
|  |  | Youth withdrawn/ depressed | 53.58 (5.83) [50 - 97] | "Refuses to talk" |
| Youth Anthropometrics (Modified from PhenX) | Recorded by experimenter | Body mass index (BMI = (Weight (lbs) × 703) ÷ (Height (in)^2^) | 18.90 (4.10) [13.13 – 32.97] | N/A |
| Youth Screen Time Survey | Youth | Mature content exposure | 1.47 (0.62) [1 - 4] | "How often do you play mature-rated video games (e.g., Call of Duty, Grand Theft Auto, Assassin's Creed, etc.)?" |
| ***Ecological Domain: Family*** *(n = 18 predictors)* | | | | |
| Parent Acculturation Survey (Modified from PhenX) | Caregiver | Caregiver bilingualism | Bilingual:  *N* = 3,138  Monolingual:  *N* = 6,252 | "Besides English, do you speak or understand another language or dialect?" |
| Parental Monitoring Survey | Youth | Parental monitoring | 4.39 (0.52) [1 - 5] | "How often do your parents/guardians know where you are?" |
| Parent Adult Self Report Survey T-Scores | Caregiver | Caregiver ADHD | 53.26 (5.60) [50 - 98] | "I am impulsive or act without thinking" |
|  |  | Caregiver aggressive behavior | 53.36 (5.07) [50 - 89] | "I physically attack people" |
|  |  | Caregiver antisocial symptoms | 53.04 (4.71) [50 - 83] | "I don't feel guilty after doing something I shouldn't" |
|  |  | Caregiver anxious/ depressed | 53.48 (5.73) [50 - 98] | "I feel worthless or inferior" |
|  |  | Caregiver attention problems | 53.93 (5.93) [50 - 94] | "I daydream a lot" |
|  |  | Caregiver hyperactivity | 52.04 (4.28) [50 - 80] | "I have trouble sitting still" |
|  |  | Caregiver inattention | 54.31 (6.21) [50 - 90] | "I am not good at details" |
|  |  | Caregiver rule-breaking behavior | 52.60 (4.75) [50 - 83] | "I lie or cheat" |
|  |  | Caregiver somatic complaints | 54.83 (6.21) [50 - 95] | "Nausea, feel sick" |
|  |  | Caregiver thought problems | 52.95 (5.11) [50 - 95] | "I have thoughts that other people would think are strange" |
|  |  | Caregiver withdrawn/ depressed | 52.86 (5.12) [50 - 97] | "There is very little I enjoy" |
| Children's Report of Parental Behavioral Inventory | Youth | Caregiver warmth (averaged between both caregivers when applicable) | 2.74 (0.29) [1 - 3] | "First caregiver (caregiver participating in study/completing protocol). Is able to make me feel better when I am upset." |
| Parent Family History Summary Scores | Caregiver | History of alcohol abuse (caregiver) | Problem endorsed by ≥ 1 caregiver:  *N* = 1,421  No problem endorsed:  *N* = 8,046 | "Mother alcohol problem" |
|  |  | History of drug abuse (caregiver) | Problem endorsed by ≥ 1 caregiver:  *N* = 2,953  No problem endorsed:  *N* = 6,351 | "Father drug problem" |
|  |  | History of suicide attempt or completion (any family member) | 0.21 (0.55) [0 - 6] | "Father attempted or committed suicide" |
| Youth Family Environment Scale, Family Conflict Subscale (Modified from PhenX) | Youth | Family conflict | 1.98 (1.93) [0 - 9] | "Family members often criticize each other." |
| ***Ecological Domain: School/Peer*** *(n = 4 predictors)* | | | | |
| School Risk and Protective Factors Survey | Youth | Positive school involvement | 13.05 (2.37) [4 - 16] | "There are lots of chances to be part of class discussions or activities." |
|  |  | School disengagement | 3.75 (1.46) [2 - 8] | "Usually, school bores me." |
|  |  | Positive view of school environment | 19.92 (2.83) [6 - 24] | "My teacher(s) notices when I am doing a good job and lets me know about it." |
| ABCD Other Resilience | Youth | Number of close friends | 5.98 (5.85) [0 - 35] | "How many close friends that are girls do you have?" |
| ***Ecological Domain: Neighborhood*** *(n = 11 predictors)* | | | | |
| Residential history derived scores | Linked external data (see Fan et al., 2021) | Median gross rent in census block | $1,111 (373.44) [$166 - $2,001] | N/A |
|  |  | Median home value in census block | $264,141 (187,274.20) [$15,400 - $1,000,001] | N/A |
|  |  | Median monthly mortgage in census block | $1,456 (603.57) [$206 - $4,001] | N/A |
|  |  | Daily estimate of NO_2_ (1-km^2^ around residence; parts per billion) | 1.73 (1.11) [0.03 - 8.93] | N/A |
|  |  | Proximity to a major road or highway (meters) | 1,161.50 (1,1237.64) [0.01 - 34,314.61] | N/A |
|  |  | Residential crowding in census block (% of housing units with >1 person per room) | 3.82 (6.52) [0 - 65.45] | N/A |
|  |  | Risk of lead exposure in census block  (% of homes at risk) | 21.44 (16.12) [0.05 - 64.54] | N/A |
|  |  | Total crimes in census block (3-year average) | 55,424 (88,723.89) [0 - 348,049] | N/A |
|  |  | Urbanicity | Urban:  *N =* 8,295  Suburban:  *N* = 277  Rural:  *N* = 768 | N/A |
|  |  | Violent crimes in census block (3-year average) | 3,873.20 (8,092.35) [0 – 31,135] | N/A |
| Youth Neighborhood Safety/Crime Survey (Modified from PhenX) | Youth | Perceived neighborhood safety | 4.02 (1.10) [1 - 5] | "My neighborhood is safe from crime." |

## Table S4.

*Model comparison of k-class latent profile models (clustered by study site; N = 9,839).*

| Model | Par | LL | Entropy | AIC | BIC | Class size |
| --- | --- | --- | --- | --- | --- | --- |
| 1-profile | 20 | -44636.48 | – | 89312.96 | 89456.85 | 9839 |
| 2-profiles | 31 | -43699.49 | 0.763 | 87460.97 | 87683.99 | 3315, 6524 |
| 3-profiles | 42 | -43309.45 | 0.670 | 86702.90 | 87005.05 | 2365, 3653, 3821 |
| 4-profiles | 53 | -43135.75 | 0.703 | 86377.49 | 86758.78 | 287, 2225, 3611, 3716 |
| 5-profiles | 64 | -42973.16 | 0.683 | 86074.31 | 86534.73 | 275, 1440, 2110, 2618, 3396 |

*Note.* Par = Number of parameters. LL = Log likelihood. AIC = Akaike Information Criterion. BIC = Bayesian Information Criterion. Class sizes are based on participants’ most likely latent profile membership.

## Table S5.

*Model comparison of k-class latent profile models (not clustered by study site; N = 9,852).*

| Model | Par | LL | Entropy | AIC | BIC | LMR  (*p*-value) | Class size |
| --- | --- | --- | --- | --- | --- | --- | --- |
| 1-profile | 20 | -44668.89 | – | 89377.77 | 89521.68 | – | 9852 |
| 2-profile | 31 | -43728.53 | 0.763 | 87519.07 | 87742.13 | 1880.70  (*p* < 0.001) | 3327, 6525 |
| 3-profile | 42 | -43337.91 | 0.670 | 86759.82 | 87062.03 | 781.25  (*p* < 0.001) | 2373, 3656, 3823 |
| 4-profile | 53 | -43164.19 | 0.703 | 86434.38 | 86815.73 | 334.05  (*p* < 0.001) | 287, 2235, 3610, 3720 |
| 5-profile | 64 | -43001.81 | 0.683 | 86131.63 | 86592.13 | 321.57  (*p* < 0.001) | 277, 1446, 2113, 2615, 3401 |

*Note.* Par = Number of parameters. LL = Log likelihood. AIC = Akaike Information Criterion. BIC = Bayesian Information Criterion. LMR = Lo-Mendell Rubin likelihood ratio test. Class sizes are based on participants’ most likely latent profile membership. Unclustered latent profile analysis was used to assess model fit via LMR, but the clustered model is used in all other analyses. The overall analytic size for unclustered analyses includes 13 additional participants who were excluded from clustered analyses for missing a valid site ID.

## Table S6*.*

*Profile-level descriptive statistics for indicators of cognitive functioning.*

|  | **Advantaged** | **Average-Average** | **Cognitive Resilience** | **Cognitive Vulnerability** |  |
| --- | --- | --- | --- | --- | --- |
| **Task** | *M(SD)* | *M(SD)* | *M(SD)* | *M(SD)* | *Test statistics and effect size* |
| Oral Reading Recognition | 92.94 (5.96) | 91.80 (6.40) | 87.60 (6.93) | 80.79 (7.22) | *F*(3,9715) = 582.3,  *p* < 0.001,  η^2^ = 0.15 |
| Picture Vocabulary | 87.63 (7.11) | 85.60 (7.41) | 79.02 (7.01) | 74.00 (7.09) | *F*(3,9727) = 886.5,  *p* < 0.001,  η^2^ = 0.21 |
| List Sorting Working Memory | 100.07 (10.85) | 98.08 (10.87) | 91.36 (12.06) | 77.67 (12.83) | *F*(3,9685) = 563.1,  *p* < 0.001,  η^2^ = 0.15 |
| WISC-V Matrix Reasoning | 10.65 (2.78) | 10.13 (2.81) | 8.58 (2.85) | 6.00 (2.88) | *F*(3,9631) = 431.1,  *p* < 0.001,  η^2^ = 0.12 |
| Flanker | 95.42 (7.93) | 95.45 (7.63) | 92.14 (9.36) | 71.90 (9.68) | *F*(3,9721) = 799.4,  *p* < 0.001,  η^2^ = 0.20 |
| Dimensional Change Card Sort | 94.48 (8.42) | 93.59 (8.23) | 89.95 (9.23) | 72.26 (11.69) | *F*(3,9721) = 670.5,  *p* < 0.001,  η^2^ = 0.17 |
| Pattern Comparison Processing Speed | 89.64 (14.43) | 88.68 (14.03) | 85.72 (14.36) | 72.14 (14.00) | *F*(3,9703) = 154.7,  *p* < 0.001,  η^2^ = 0.05 |
| Picture Sequence Memory | 105.23 (11.94) | 103.31 (11.76) | 99.32 (11.46) | 90.41 (9.39) | *F*(3,9716) = 226.1,  *p* < 0.001,  η^2^ = 0.07 |
| RAVLT Short Delay | 10.31 (2.86) | 9.89 (2.85) | 8.66 (3.08) | 5.78 (3.32) | *F*(3,9620) = 286.6,  *p* < 0.001,  η^2^ = 0.08 |
| RAVLT Long Delay | 9.83 (3.09) | 9.46 (3.02) | 8.12 (3.20) | 5.39 (3.23) | *F*(3,9664) = 316.6,  *p* < 0.001,  η^2^ = 0.09 |

*Note*. Total *N*=9,839; Advantaged (ADV) *n=*3,716; Average-Average (AVG) *n=*3,611; Cognitive Resilience (CR) *n=*2,225; Cognitive Vulnerability (CV) *n* =287. M = Mean. SD = Standard deviation. WISC-V = Weschsler Intelligence Scale for Children: 5^th^ edition. RAVLT = Rey Auditory Verbal Learning Test. Means for NIH toolbox tasks were calculated using raw, uncorrected scores. All profiles differed across all cognitive task scores, except for ADV and AVG where there were no significant differences in performance on the Flanker Task (*p* = 1.00). Significance was tested through ANOVA tests.

## Table S7.

*Profile-level WISC-V Matrix Reasoning scores and GPA for LPA validation.*

| Profile | WISC-V Matrix Reasoning (Baseline) | | GPA  (2-year follow-up) | |
| --- | --- | --- | --- | --- |
|  | Mean (SD) | *F* (η^2^) | Mean (SD) | *F* (η^2^) |
| Advantaged (ADV) | 10.65 (2.78) | 497.30 (0.13) | 3.54 (0.45) | 225.80 (0.08) |
| Average-Average (AVG) | 10.13 (2.81) |  | 3.41 (0.58) |  |
| Cognitive Resilience (CR) | 8.58 (2.85) |  | 3.17 (0.71) |  |
| Cognitive Vulnerability (CV) | 6.00 (2.88) |  | 2.85 (0.81) |  |

*Note.* Significant differences between profiles were determined via ANOVA and eta-squared (η^2^) was used to gauge the effect size. Both WISC-V Matrix Reasoning and GPA significantly differed between profiles (i.e., *p* < 0.001). WISC-V = Weschsler Intelligence Scale for Children: 5^th^ edition. GPA = Grade point average.

## Table S8.

*Model fit statistics for post-hoc tests of measurement invariance across demographic groups.*

| **Grouping variable** | **Model** | **AIC** | **BIC** | **LL** | **∆AIC** | **∆BIC** | **∆LL** |
| --- | --- | --- | --- | --- | --- | --- | --- |
| Race-ethnicity | Scalar | 103266.6 | 104083.1 | -51518.3 | 2027.514 | 1601.523 | -1073.76 |
|  | Configural | 101239.1 | 102481.6 | -50444.5 |  |  |  |
| Gender | Scalar | 96908.98 | 97384.58 | -48387.5 | 122.716 | -19.254 | -81.358 |
|  | Configural | 96786.26 | 97403.83 | -48306.1 |  |  |  |
| Family Structure | Scalar | 92752.43 | 93227.65 | -46309.2 | 772.669 | 630.811 | -406.334 |
|  | Configural | 91979.76 | 92596.84 | -45902.9 |  |  |  |
| Country of origin | Scalar | 87413.25 | 87888.99 | -43639.6 | 12.182 | -129.831 | -26.091 |
|  | Configural | 87401.06 | 88018.82 | -43613.5 |  |  |  |

*Note.* Model fit indices are reported for unconstrained (configural) and fully constrained (scalar) models for each demographic grouping variable. ΔAIC, ΔBIC, and ΔLL values reflect the difference between scalar and configural models (scalar – configural). Higher positive values indicate worse fit for the scalar model, suggesting potential non-invariance. AIC = Akaike information criterion. BIC = Bayesian information criterion. LL = Log-likelihood.

## Table S9.

*Odds ratios for the ecological predictors of profile membership in Cognitive Resilience (CR) versus Cognitive Vulnerability (CV).*

| **Variable** | **OR [95% CI]** |
| --- | --- |
| ***Ecological Domain: Individual*** |  |
| Pubertal development score | 1.201* [1.167-1.236] |
| Bilingual | 1.144* [1.124-1.165] |
| Prosocial behavior | 1.040* [1.031-1.050] |
| Psychosis symptoms | 1.026* [1.023-1.031] |
| Age | 1.025* [1.022-1.030] |
| Withdrawn symptoms | 1.010* [1.009-1.010] |
| Behavioral activation (BAS) | 1.009* [1.007-1.010] |
| Rule breaking | 1.005* [1.004-1.006] |
| Body mass index | 1.002 [0.997-1.007] |
| Impulsivity: Negative urgency | 1.002 [0.997-1.007] |
| Somatic symptoms | 1.002* [1.000-1.003] |
| Behavioral inhibition (BIS) | 0.999 [0.996-1.002] |
| Aggression | 0.998* [0.997-0.999] |
| Thought problems | 0.992* [0.991-0.993] |
| Anxiety/depression | 0.991* [0.991-0.992] |
| Impulsivity: Positive urgency | 0.983* [0.979-0.987] |
| Social issues | 0.983* [0.980-0.984] |
| Attention problems | 0.981* [0.980-0.982] |
| Impulsivity: Sensation seeking | 0.978* [0.976-0.983] |
| Exposure to mature content | 0.977 [0.947-1.003] |
| Impulsivity: Lack of premeditation | 0.973* [0.968-0.980] |
| Impulsivity: Lack of perseverance | 0.969* [0.961-0.976] |
| Monolingual | 0.874* [0.858-0.889] |
| ***Ecological Domain: Family*** |  |
| Bilingual caregiver(s) | 1.133* [1.118-1.161] |
| Caregiver monitoring | 1.101* [1.071-1.151] |
| Caregiver thought problems | 1.022* [1.023-1.023] |
| Caregiver somatic symptoms | 1.017* [1.016-1.018] |
| Caregiver withdrawn symptoms | 1.016* [1.015-1.016] |
| Caregiver rule breaking | 1.009* [1.007-1.011] |
| Caregiver warmth | 1.008 [0.958-1.044] |
| No caregiver alcohol abuse | 1.008 [0.985-1.037] |
| Caregiver attention problems | 1.004* [1.004-1.005] |
| Caregiver drug abuse | 1.003 [0.980-1.021] |
| Caregiver inattention | 1.002* [1.002-1.003] |
| Caregiver ADHD | 1.000* [1.000-1.000] |
| Caregiver anxiety/depression | 0.999* [0.998-0.999] |
| Caregiver aggression | 0.998* [0.997-0.998] |
| No caregiver drug abuse | 0.997 [0.980-1.020] |
| Caregiver antisocial symptoms | 0.994* [0.993-0.994] |
| Caregiver hyperactivity | 0.992* [0.991-0.993] |
| Caregiver alcohol abuse | 0.992 [0.965-1.015] |
| Family history of suicide | 0.970* [0.928-0.998] |
| Family conflict | 0.970* [0.967-0.971] |
| Monolingual caregiver(s) | 0.882* [0.861-0.894] |
| ***Ecological Domain: School/Peer*** |  |
| Positive school involvement | 0.986* [0.979-0.991] |
| Number of close friends | 0.982* [0.978-0.982] |
| Positive view of the school environment | 0.980* [0.976-0.983] |
| School disengagement | 0.944* [0.933-0.953] |
| ***Ecological Domain: Neighborhood*** |  |
| Suburban CB | 1.304* [1.230-1.381] |
| Residential crowding in CB | 1.097* [1.084-1.101] |
| NO_2_ (1 km^2^ around residence) | 1.034* [1.023-1.044] |
| Risk of lead exposure in CB | 1.009* [1.008-1.010] |
| Urban CB | 1.001 [0.950-1.024] |
| Violent crime in CB | 1.000* [1.000-1.000] |
| Total crime in CB | 1.000* [1.000-1.000] |
| Median home value in CB | 1.000* [1.000-1.000] |
| Proximity to roads from residence | 1.000* [1.000-1.000] |
| Median monthly mortgage in CB | 0.999* [0.999-0.999] |
| Median gross rent in CB | 0.999* [0.999-0.999] |
| Rural CB | 0.907* [0.877-0.938] |
| Perceived neighborhood safety | 0.879* [0.870-0.891] |

*Note.* ORs were derived using median estimates of the bootstrapped distribution of logistic ridge regression coefficients (*b* = 1,000; see Methods). ORs greater than 1 indicate that a 1-unit increase in the variable (if continuous) or an endorsement of the trait (if categorical) increased youths’ odds of being in the CR profile over the CV profile. ORs less than one indicate that the variable decreased youths’ odds of being in the CR profile over the CV profile. Asterisks (*) indicate predictors deemed to be significant by examining whether the 95% CI of the OR includes 1. Due to rounding in this table, statistically significant predictors may have an OR of 1.000 (e.g., 95% CI = 1. 00000026‒1.00000045).

## Table S10.

*Languages spoken among bilingual youth in the analytic sample.*

| **Language** | **Proportion of bilingual youth in the analytic sample** |
| --- | --- |
| Spanish | 70.54% |
| French | 5.02% |
| Mandarin | 4.76% |
| “Other” | 3.13% |
| Japanese | 1.39% |
| American Sign Language (ASL) | 1.47% |
| Russian | 1.08% |
| All other languages | 12.61% |

*Note*. Total *n* bilingual in the analytic sample = 3,805. A total of 44 languages were endorsed at least once. If participants endorsed “[speaking] or [understanding]” a language other than English, they were asked, “What other language or dialect do you speak or understand (besides English)?”.

# Supplementary Figures

## Figure S1.

*Correlations between cognitive domain factor scores at baseline and task performance on randomly selected cognitive tasks administered at the 2-year follow-up.*

*
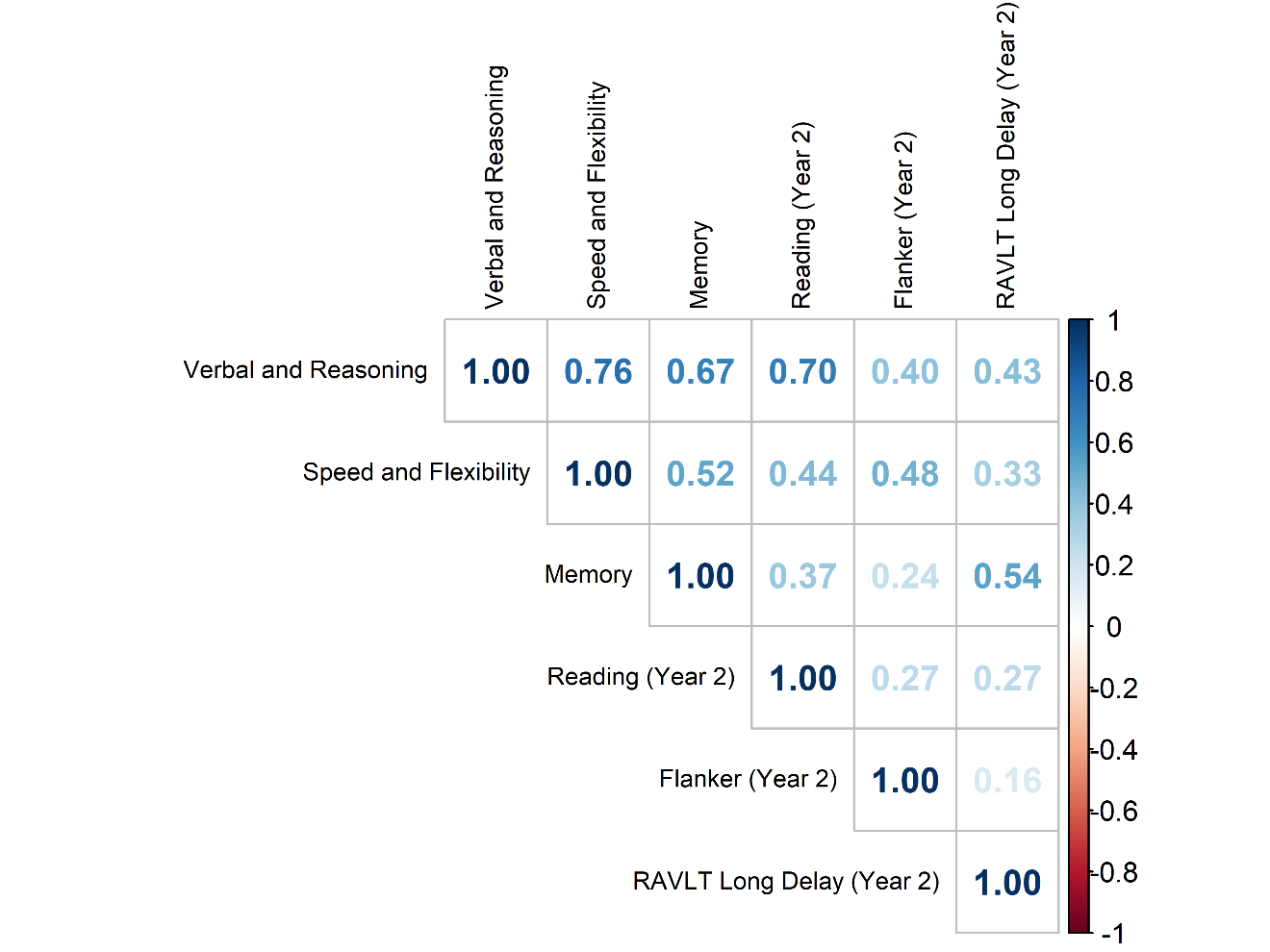
*

*Note.* RAVLT = Rey Auditory Verbal Learning Task. To validate the structure of the 3-class cognitive factor structure, we correlated factor scores with youths’ task performance on one randomly selected task per cognitive domain, collected approximately two years after baseline data collection (*N* = 6,509 youth with follow-up data). As expected, performance on these randomly selected tasks at Year 2 was most strongly correlated with the cognitive domain each task had been fit onto in the baseline CFA. Specifically, the strongest correlations for each cognitive domain were the NIHTB Oral Reading Recognition Task with Verbal and Reasoning (*r* = 0.70), the NIHTB Flanker Task with Speed and Flexibility (*r* = 0.48), and the RAVLT Long Delay with Memory (*r* = 0.54). All correlations were statistically significant (*p* < 0.001).

## Figure S2.

*Ecological predictors of profile membership in Cognitive Resilience (CR) vs. Average-Average (AVG).*


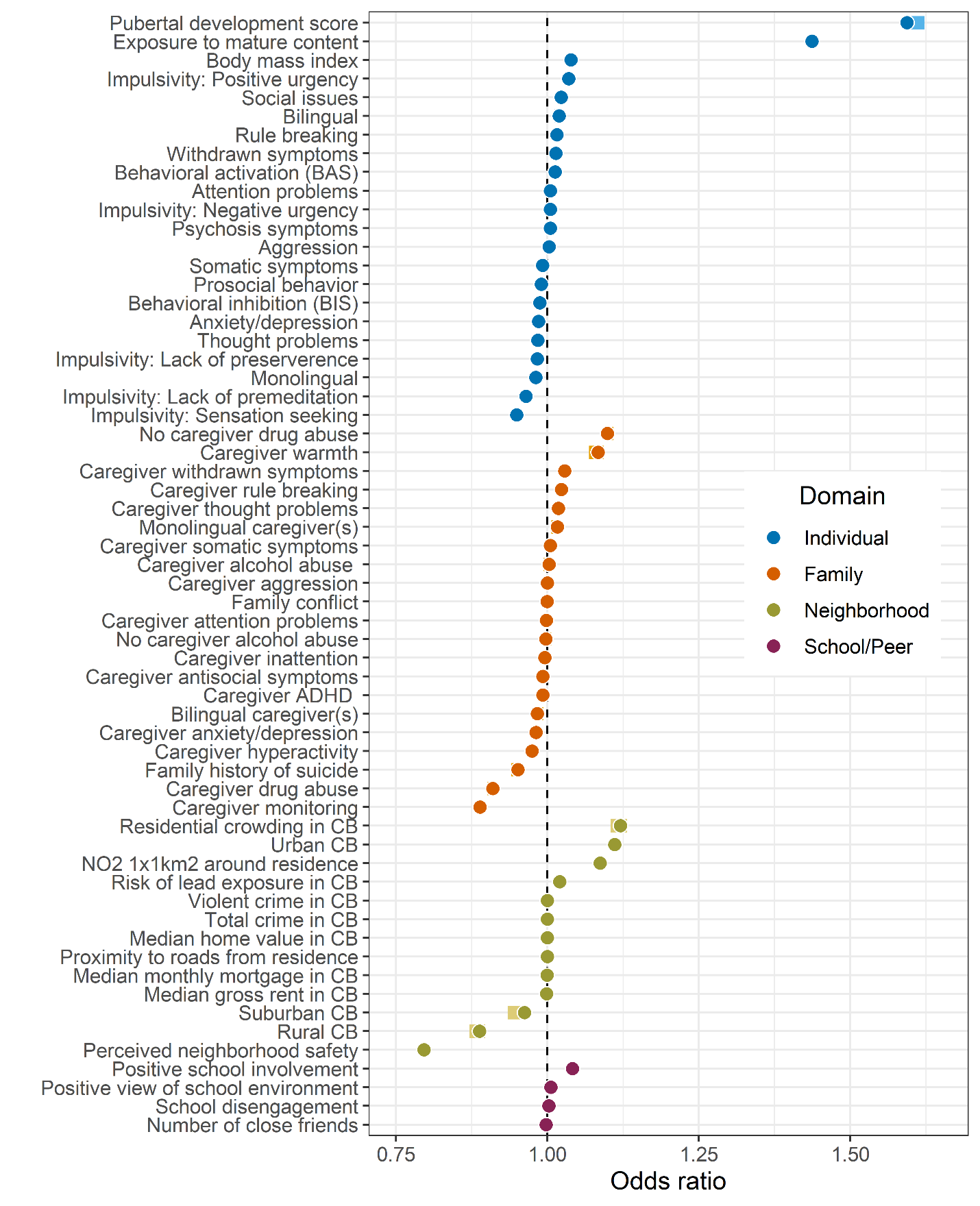


*Note.* Total *N* = 9,839.

## Figure S3.

*Ecological predictors of profile membership in Cognitive Resilience (CR) vs. Advantaged (ADV)*
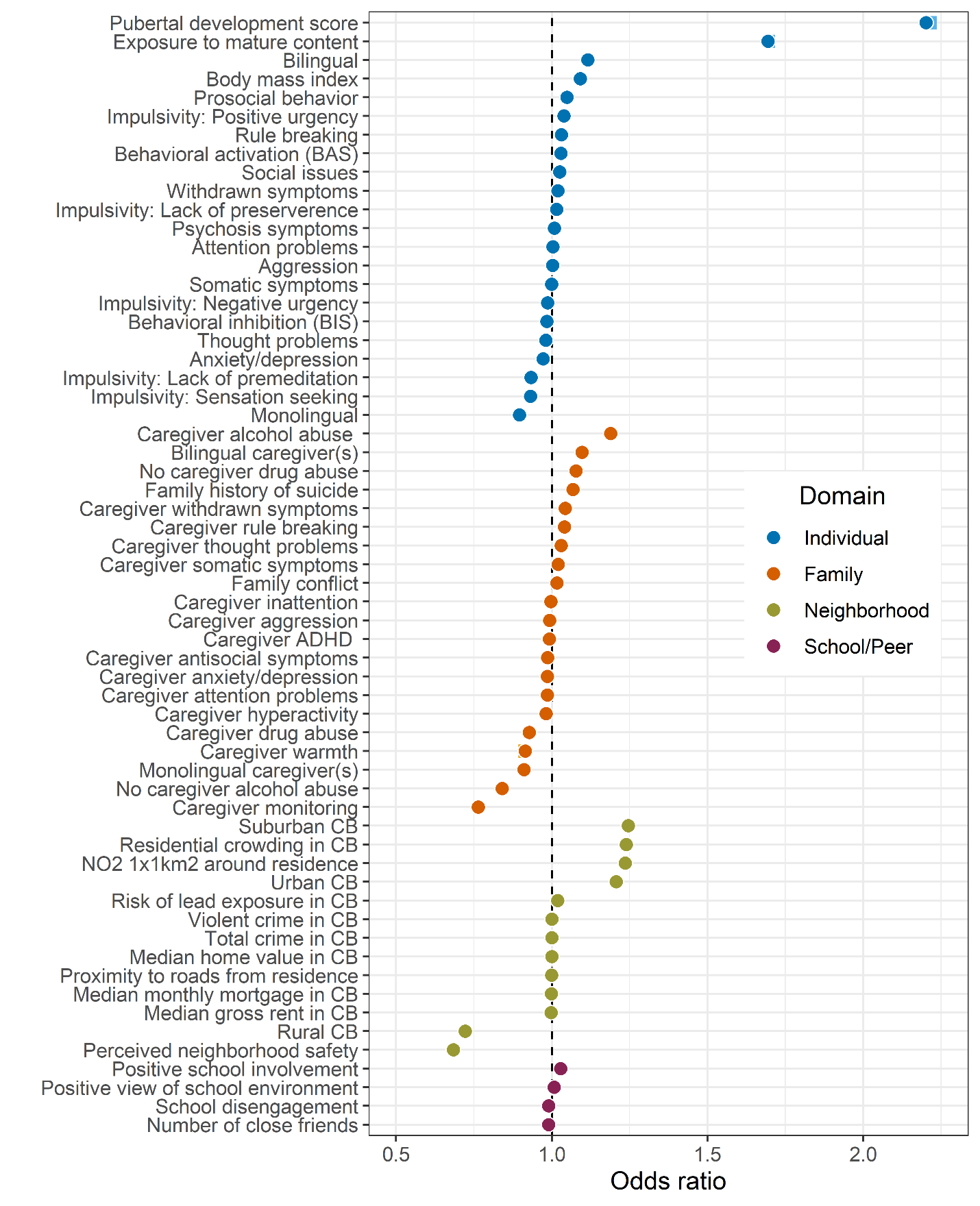


*Note.* Total *N* = 9,839.

## Figure S4.

*Ecological predictors of profile membership in Cognitive Vulnerability (CV) vs. Average-Average (AVG).*


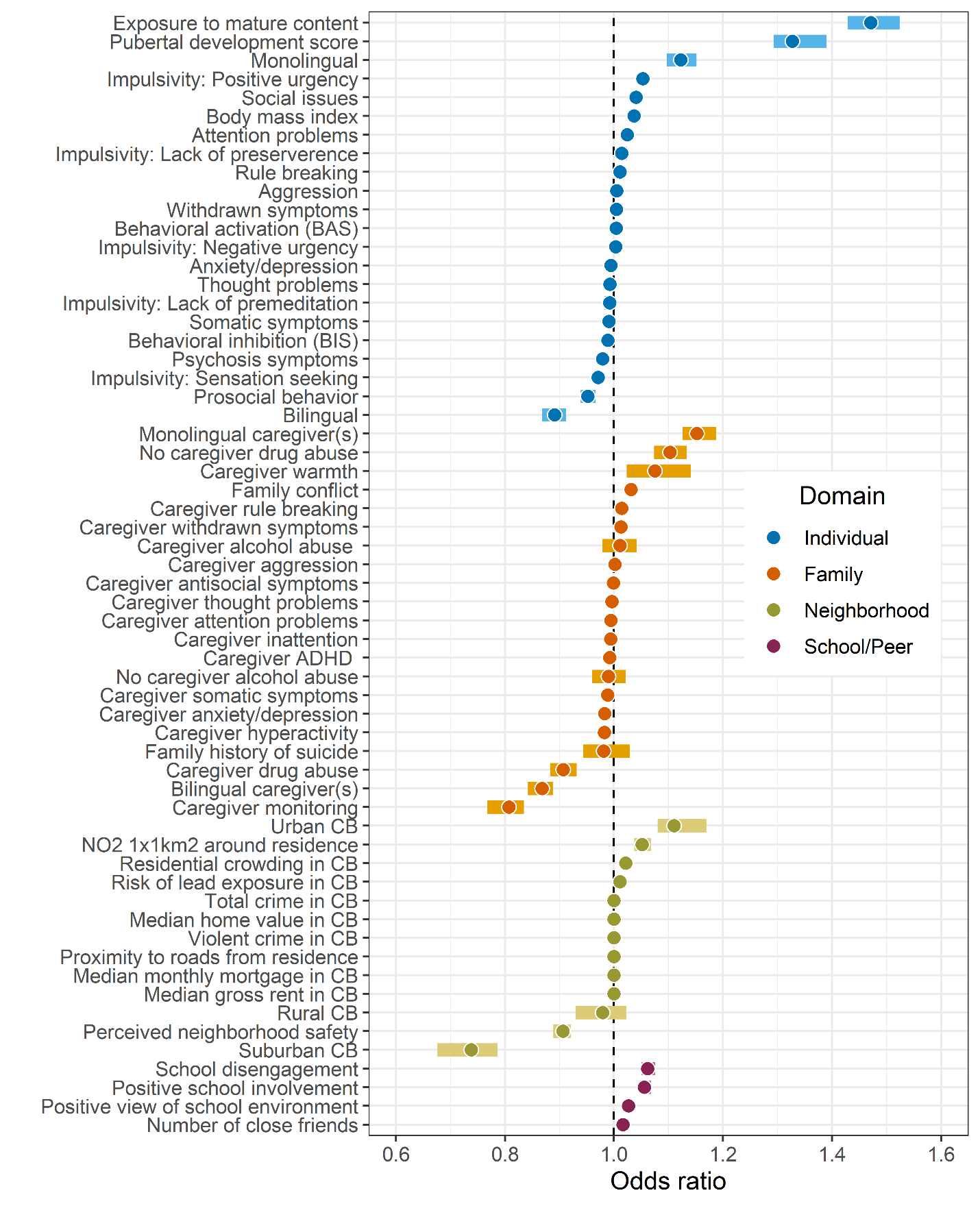


*Note.* Total *N* = 9,839

## Figure S5.

*Ecological predictors of profile membership in Cognitive Vulnerability (CV) vs. Advantaged (ADV).*


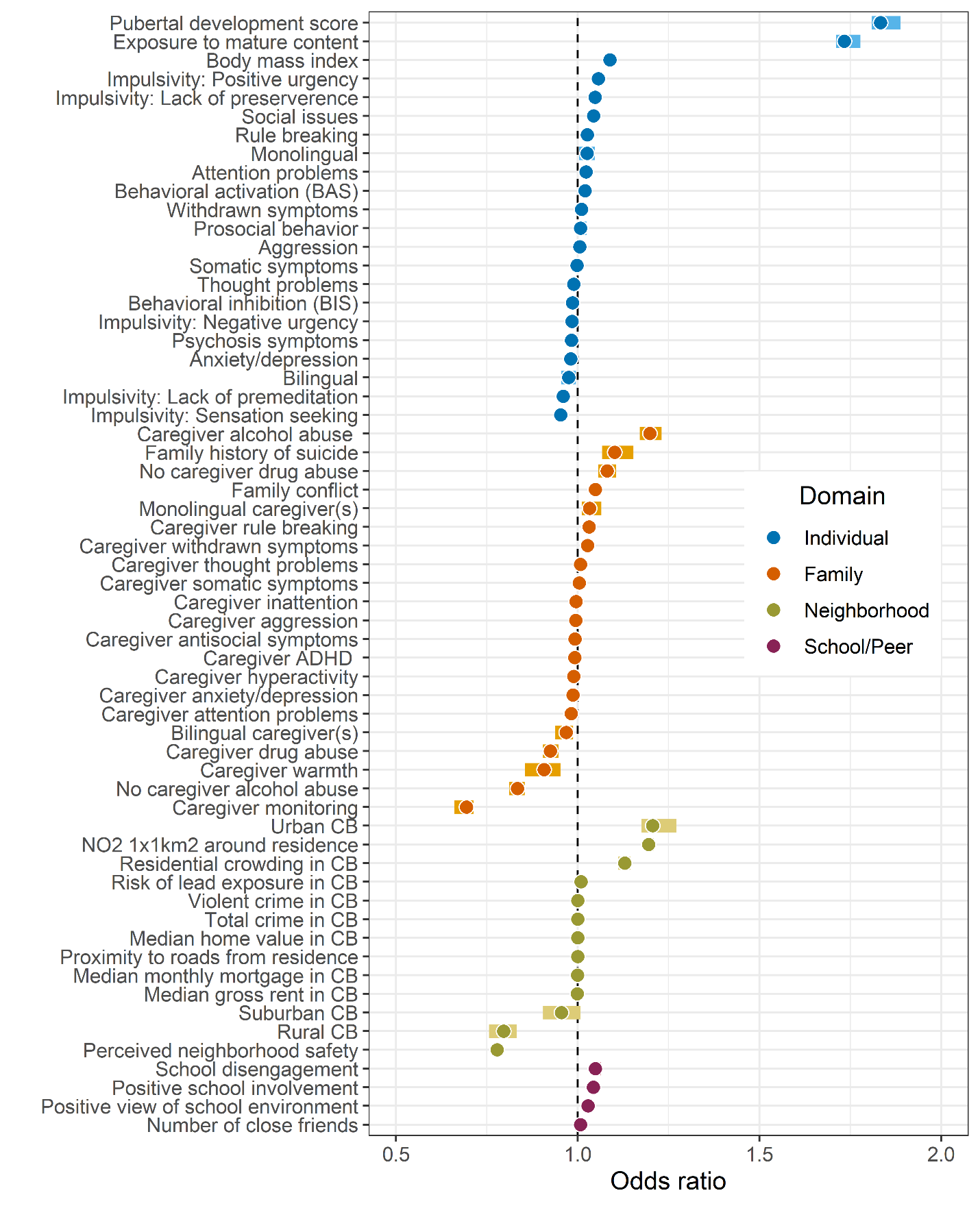


*Note.* Total *N* = 9,839.

## Figure S6.

*Ecological predictors of profile membership in Average-Average (AVG) vs. Advantaged (ADV).*

*
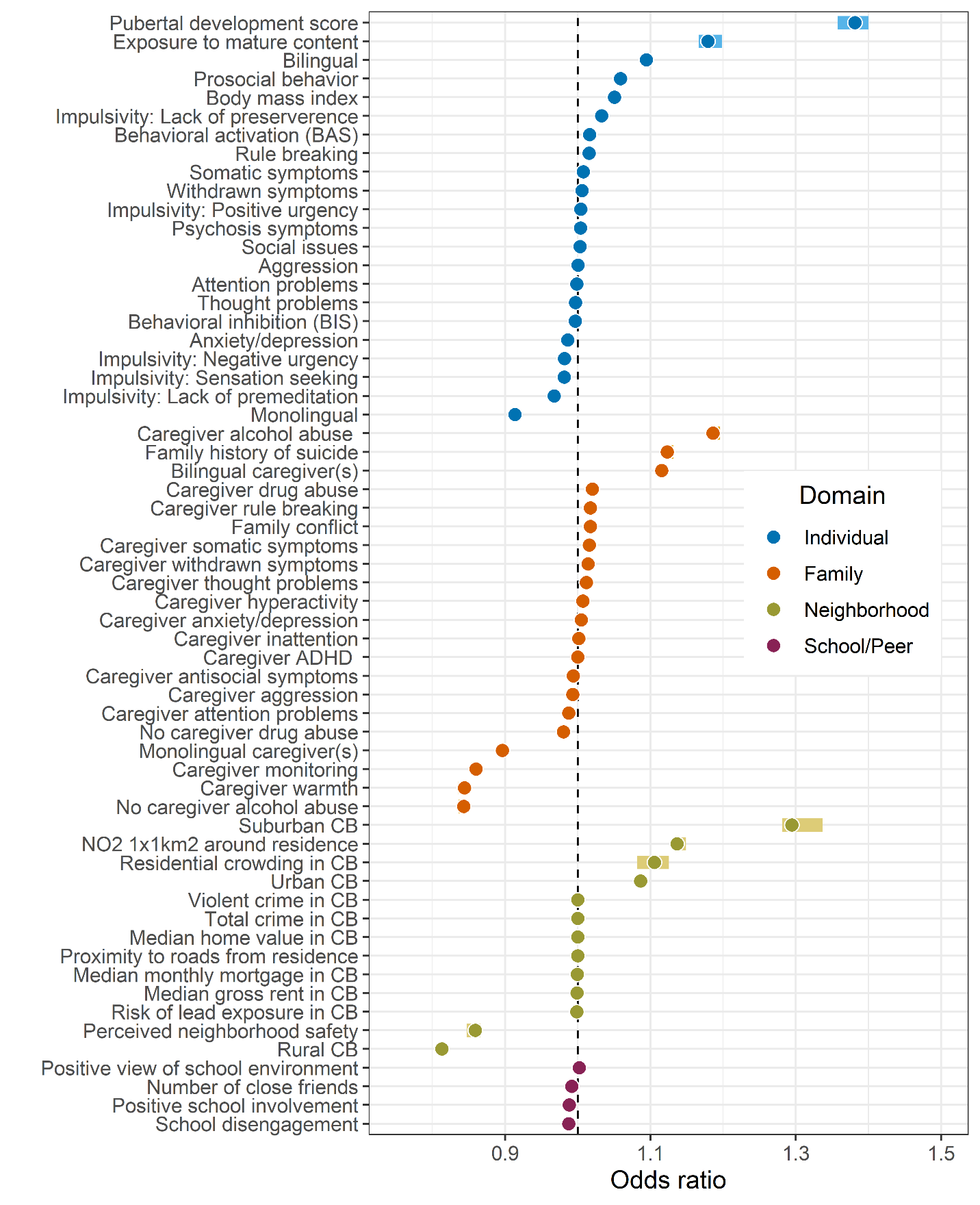
*

*Note.* Total *N* = 9,839.

## Figure S7.

Violin plot depicting profile-level distributions for standardized factor scores of socioeconomic resources (SER) and cognitive domain scores.


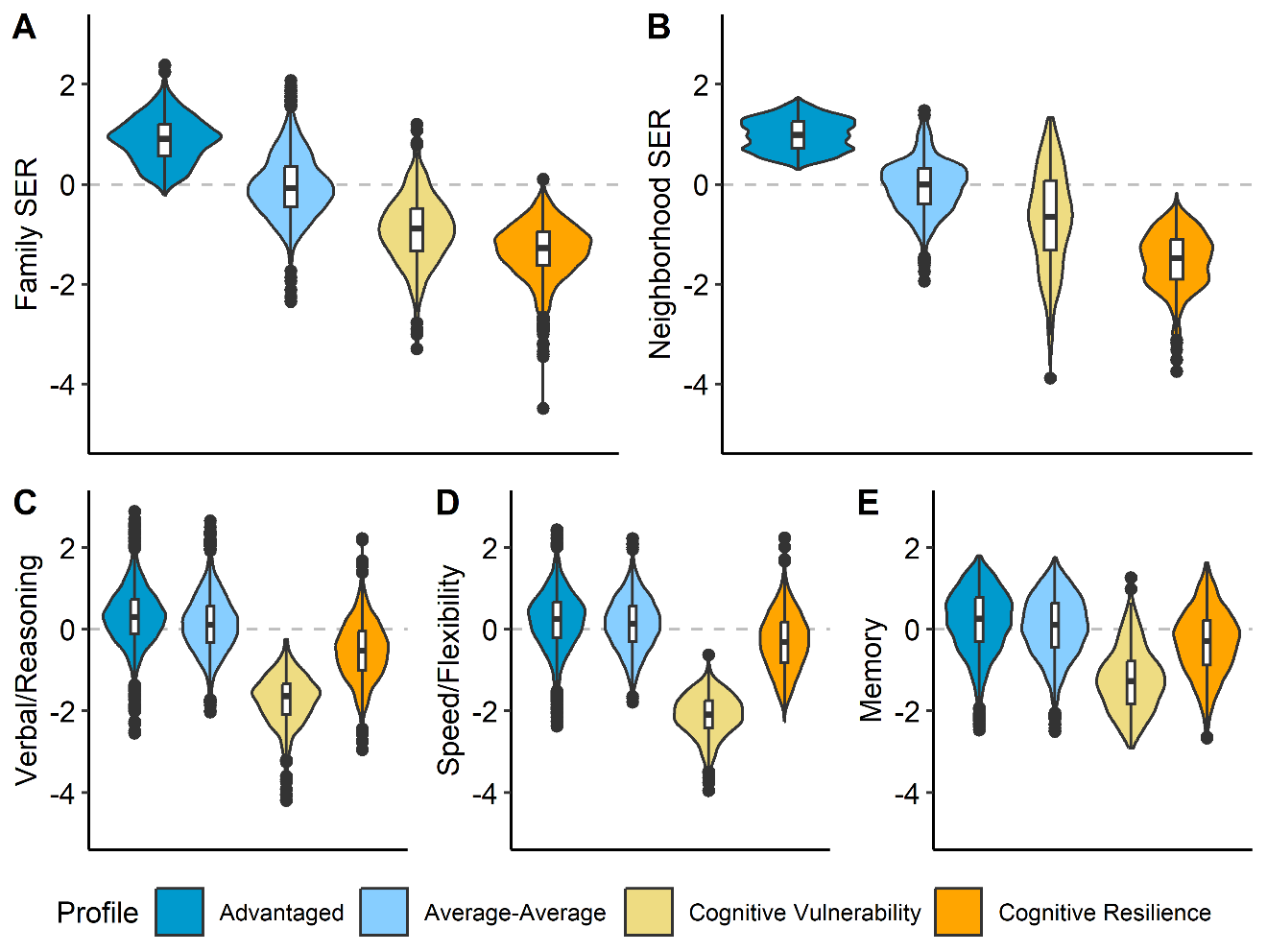


*Note.* Total *N* = 9,839. Cognitive Resilience *n* = 2,225. Cognitive Vulnerability *n* = 287. Average-Average *n* = 3,611. Advantaged *n* = 3,716. See Table 1 and Table S6 for descriptive statistics for unstandardized SER and cognitive task variables, respectively.

# References

Bauer, P. J., Dikmen, S. S., Heaton, R. K., Mungas, D., Slotkin, J., & Beaumont, J. L. (2013). Iii. Nih Toolbox Cognition Battery (cb): Measuring Episodic Memory. *Monographs of the Society for Research in Child Development*, *78*(4), 34–48. https://doi.org/10.1111/mono.12033

Carlozzi, N. E., Beaumont, J. L., Tulsky, D. S., & Gershon, R. C. (2015). The NIH Toolbox Pattern Comparison Processing Speed Test: Normative Data. *Archives of Clinical Neuropsychology*, *30*(5), 359–368. https://doi.org/10.1093/arclin/acv031

Dikmen, S. S., Bauer, P. J., Weintraub, S., Mungas, D., Slotkin, J., Beaumont, J. L., Gershon, R., Temkin, N. R., & Heaton, R. K. (2014). Measuring Episodic Memory Across the Lifespan: NIH Toolbox Picture Sequence Memory Test. *Journal of the International Neuropsychological Society*, *20*(6), 611–619. https://doi.org/10.1017/S1355617714000460

Eriksen, B. A., & Eriksen, C. W. (1974). Effects of noise letters upon the identification of a target letter in a nonsearch task. *Perception & Psychophysics*, *16*(1), 143–149. https://doi.org/10.3758/BF03203267

Fan, C. C., Marshall, A., Smolker, H., Gonzalez, M. R., Tapert, S. F., Barch, D. M., Sowell, E., Dowling, G. J., Cardenas-Iniguez, C., Ross, J., Thompson, W. K., & Herting, M. M. (2021). Adolescent Brain Cognitive Development (ABCD) study Linked External Data (LED): Protocol and practices for geocoding and assignment of environmental data. *Developmental Cognitive Neuroscience*, *52*, 101030. https://doi.org/10.1016/j.dcn.2021.101030

Garavan, H., Bartsch, H., Conway, K., Decastro, A., Goldstein, R. Z., Heeringa, S., Jernigan, T., Potter, A., Thompson, W., & Zahs, D. (2018). Recruiting the ABCD sample: Design considerations and procedures. *Developmental Cognitive Neuroscience*, *32*, 16–22. https://doi.org/10.1016/j.dcn.2018.04.004

Gershon, R. C., Cook, K. F., Mungas, D., Manly, J. J., Slotkin, J., Beaumont, J. L., & Weintraub, S. (2014). Language Measures of the NIH Toolbox Cognition Battery. *Journal of the International Neuropsychological Society*, *20*(6), 642–651. https://doi.org/10.1017/S1355617714000411

Guadagnoli, E., & Velicer, W. F. (1988). Relation of sample size to the stability of component patterns. *Psychological Bulletin*, *103*(2), 265–275. <https://doi.org/10.1037/0033-2909.103.2.265>

Roth, B., Becker, N., Romeyke, S., Schäfer, S., Domnick, F., & Spinath, F. M. (2015). Intelligence and school grades: A meta-analysis. *Intelligence*, *53*, 118–137. <https://doi.org/10.1016/j.intell.2015.09.002>

Schmidt, M. (1996). *Rey auditory verbal learning test* (pp. 1-125). Los Angeles: Western Psychological Services.

Selvitopu, A., & Kaya, M. (2023). A Meta-Analytic Review of the Effect of Socioeconomic Status on Academic Performance. *Journal of Education*, *203*(4), 768–780. <https://doi.org/10.1177/00220574211031978>

Sripada, C., Angstadt, M., Taxali, A., Clark, D. A., Greathouse, T., Rutherford, S., Dickens, J. R., Shedden, K., Gard, A. M., Hyde, L. W., Weigard, A., & Heitzeg, M. (2021). Brain-wide functional connectivity patterns support general cognitive ability and mediate effects of socioeconomic status in youth. *Translational Psychiatry*, *11*(1), 571. https://doi.org/10.1038/s41398-021-01704-0

Taylor, R. L., Cooper, S. R., Jackson, J. J., & Barch, D. M. (2020). Assessment of Neighborhood Poverty, Cognitive Function, and Prefrontal and Hippocampal Volumes in Children. *JAMA Network Open*, *3*(11), e2023774. https://doi.org/10.1001/jamanetworkopen.2020.23774

Weintraub, S., Dikmen, S. S., Heaton, R. K., Tulsky, D. S., Zelazo, P. D., Bauer, P. J., Carlozzi, N. E., Slotkin, J., Blitz, D., Wallner-Allen, K., Fox, N. A., Beaumont, J. L., Mungas, D., Nowinski, C. J., Richler, J., Deocampo, J. A., Anderson, J. E., Manly, J. J., Borosh, B., … Gershon, R. C. (2013). Cognition assessment using the NIH Toolbox. *Neurology*, *80*(11 Supplement 3), S54–S64. https://doi.org/10.1212/WNL.0b013e3182872ded

Wechsler, D. (2014). Wechsler Intelligence Scale for Children (5th ed.). San Antonio, TX: NCS Pearson.

Zelazo, P. D., Anderson, J. E., Richler, J., Wallner-Allen, K., Beaumont, J. L., Conway, K. P., Gershon, R., & Weintraub, S. (2014). NIH Toolbox Cognition Battery (CB): Validation of Executive Function Measures in Adults. *Journal of the International Neuropsychological Society*, *20*(6), 620–629. https://doi.org/10.1017/S1355617714000472
